# Bystander monocytic cells drive infection-independent NLRP3 inflammasome response to SARS-CoV-2

**DOI:** 10.1128/mbio.00810-24

**Published:** 2024-09-06

**Authors:** Leon L. Hsieh, Monika Looney, Alexis Figueroa, Guido Massaccesi, Georgia Stavrakis, Eduardo U. Anaya, Franco R. D'Alessio, Alvaro A. Ordonez, Andrew S. Pekosz, Victor R. DeFilippis, Petros C. Karakousis, Andrew H. Karaba, Andrea L. Cox

**Affiliations:** 1 Department of Medicine, Johns Hopkins University School of Medicine, Baltimore, Maryland, USA; 2 W. Harry Feinstone Department of Molecular Microbiology and Immunology, Johns Hopkins University Bloomberg School of Public Health, Baltimore, Maryland, USA; 3 Department of Pediatrics, Johns Hopkins University School of Medicine, Baltimore, Maryland, USA; 4 Vaccine and Gene Therapy Institute, Oregon Health and Science University, Portland, Oregon, USA; 5 Department of International Health, Johns Hopkins Bloomberg School of Public Health, Baltimore, Maryland, USA; Washington University in St. Louis School of Medicine, St. Louis, Missouri, USA

**Keywords:** inflammasome, cytokines, SARS-CoV-2, NLRP3, macrophages

## Abstract

The pathogenesis of COVID-19 is associated with a hyperinflammatory immune response. Monocytes and macrophages play a central role in this hyperinflammatory response to SARS-CoV-2. NLRP3 inflammasome activation has been observed in monocytes of patients with COVID-19, but the mechanism and consequences of inflammasome activation require further investigation. In this study, we inoculated a macrophage-like THP-1 cell line, primary differentiated human nasal epithelial cell (hNEC) cultures, and primary monocytes with SARS-CoV-2. We found that the activation of the NLRP3 inflammasome in macrophages does not rely on viral replication, receptor-mediated entry, or actin-dependent entry. SARS-CoV-2 productively infected hNEC cultures without triggering the production of inflammasome cytokines IL-18 and IL-1β. Importantly, these cytokines did not inhibit viral replication in hNEC cultures. SARS-CoV-2 inoculation of primary monocytes led to inflammasome activation and induced a macrophage phenotype in these cells. Monocytic cells from bronchoalveolar lavage (BAL) fluid, but not from peripheral blood, of patients with COVID-19, showed evidence of inflammasome activation, expressed the proinflammatory marker CD11b, and displayed oxidative burst. These findings highlight the central role of activated macrophages, as a result of direct viral sensing, in COVID-19 and support the inhibition of IL-1β and IL-18 as potential therapeutic strategies to reduce immunopathology without increasing viral replication.

**IMPORTANCE:**

Inflammasome activation is associated with severe COVID-19. The impact of inflammasome activation on viral replication and mechanistic details of this activation are not clarified. This study advances our understanding of the role of inflammasome activation in macrophages by identifying TLR2, NLRP3, ASC, and caspase-1 as dependent factors in this activation. Further, it highlights that SARS-CoV-2 inflammasome activation is not a feature of nasal epithelial cells but rather activation of bystander macrophages in the airway. Finally, we demonstrate that two pro inflammatory cytokines produced by inflammasome activation, IL-18 and IL-1β, do not restrict viral replication and are potential targets to ameliorate pathological inflammation in severe COVID-19.

## INTRODUCTION

Coronavirus disease-19 (COVID-19), caused by SARS-CoV-2, spans mild disease with few symptoms to multiorgan failure and death (1, 2). One hallmark of severe disease appears to be immune dysregulation, characterized by elevated proinflammatory markers (such as C-reactive protein) and inflammatory cytokines (3–7), particularly in the later stages of illness. Both evasion of type 1 interferon by SARS-CoV-2 (8–10) and inflammasomes have been implicated in the pathogenesis of severe COVID-19 (7, 10–15). Activation of inflammasomes by infection or tissue damage leads to an inflammatory type of cell death called pyroptosis and the release of proinflammatory cytokines, including IL-1β and IL-18 (16). We and others have demonstrated that IL-1β and IL-18 are elevated in the plasma of patients with severe COVID-19 (7, 10, 17), and lung sections from autopsies of patients who died of COVID-19 contain increased NLRP3 specks (11, 15), consistent with inflammasome activation during SARS-CoV-2 infection. Importantly, the outcome of the production of these cytokines on viral replication and whether inflammasome activation happens at sites other than the lungs are largely unknown.

Canonical inflammasome activation begins when a pathogen-associated molecular pattern (PAMP) is recognized by a pattern recognition receptor (PPR); a Toll-like receptor (TLR) or a retinoic acid-inducible gene I (RIG-I)-like receptor (18, 19). Signaling through PRRs primes the cell by inducing the canonical nuclear factor κB (NF- κB) pathway, leading to transcriptional upregulation of inflammasome components, particularly pro-caspase-1 and pro-IL-1β. The second signal occurs when one of multiple inflammasome adaptor proteins senses the same or additional PAMP or danger-associated molecular patterns (DAMPs), such as potassium efflux or mitochondrial damage. Many of these adapter proteins come from the nucleotide-binding domain and leucine-rich repeat-containing receptors (NLRs) family of proteins, which are capable of sensing a diverse array of danger or stress signals (20, 21). Once activated, these adapter proteins lead to oligomerization and recruitment of the adaptor apoptosis-associated speck-like protein containing a caspase-recruitment domain (ASC) to form inflammasome specks. Upon oligomerization of ASC, caspase-1 (CASP-1) is recruited and activated via autocatalysis. CASP-1 then cleaves pro-IL-1β, pro-IL-18, and gasdermin-D (GSDMD) leading to GSDMD-based pores in the cellular membrane and release of active IL-1β and IL-18 (16, 22, 23). NLRP3 is a sensor central to inflammasome signaling in many viral infections, including SARS-CoV-1 (24–26). Specifically, several SARS-CoV-1 proteins, including ORF3a, ORF8b, and viral protein E, have been shown to trigger NLRP3 inflammasome signaling through multiple mechanisms (25, 27, 28). However, DAMPs, such as mitochondrial DNA, ATP, and heat shock proteins, released by dying or damaged cells are also capable of activating inflammasomes (29–32). Prior work has demonstrated that SARS-CoV-2 infected epithelial cells can indeed provide both signals 1 and 2 to inflammasome activation (14). Studies have shown that the bacterial ligand, Pam3cys, or non-neutralizing antibodies are required for SARS-CoV-2 to triggers NLRP3 inflammasome (11, 12, 15). Therefore, additional research investigating whether SARS-CoV-2 can directly activate the inflammasome in the absence of specific immunity or bacterial ligands is needed. Although IL-1β and IL-18 levels in the plasma and lungs correlate with severe COVID-19 (5, 7, 33–35), whether these inflammatory cytokines restrict viral replication and the consequence of inflammasome activation in the lung have not been clarified.

To address these knowledge gaps, we investigated the mechanism of inflammasome activation in response to SARS-CoV-2, including whether it requires viral replication, if it occurs in nasal epithelia, and the impact of these inflammatory cytokines on viral replication. In addition, we compared monocytic cells from the BAL fluid and the blood of patients with COVID-19 to investigate the site(s) of inflammasome activation. Our study demonstrates that SARS-CoV-2 directly activates inflammasomes through an NLRP3-, CASP-1-, and ASC-dependent process in human macrophages, but not in primary differentiated human nasal epithelial cell (hNEC) cultures. In primary monocytes, SARS-CoV-2 induced inflammasome activation in a small population that forms ASC specks and upregulates the M2 macrophage and alveolar macrophage marker, CD206. Monocytes and macrophages from the BAL fluid, but not from the peripheral blood of COVID-19 patients, displayed caspase-1 cleavage. These caspase-1 cleaved monocytes exhibited higher ROS production and expression of CD11b, which correlated with the dysregulation of neutrophil and monocyte composition in the airways. This study provides critical insight into the mechanism of COVID-19 inflammation and highlights the potential benefits of inhibiting macrophage-mediated inflammation.

## MATERIALS AND METHODS

### Virus

SARS-CoV-2/Wuhan-Hu-1/2020 virus (SARS-CoV-2/Wu-1; GISAID: EPI_ISL_2509960) was obtained through the U.S. Centers for Disease Control and Prevention (CDC), and SARS-CoV-2/USA/DC-HP00007/2020 (SARS-CoV-2/HP07; GISAID: EPI_ISL_434688) was isolated from a nasal swab collected from a patient at the Johns Hopkins Hospital, as described previously (36). HP07 was used for the infection of hNC cultures and Wu-1 was used for all other experiments. The virus was propagated in a Biosafety Level 3 (BSL3) laboratory in VeroE6 cells using methods described previously (37, 38). Infectious virus concentrations were determined using VeroE6-TMPRSS2 cells using a standard median tissue culture infectious dose (TCID_50_). The A/Baltimore/R0243/2018 H3N2 strain of influenza A virus (IAV) was isolated from clinical samples as described previously (39). Inactivation of SARS-CoV-2 was achieved by adding 0.05% β-propiolactone to the virus stock overnight at 4°C, followed by incubation at 37°C for 2 h to allow β-propiolactone breakdown into nontoxic by-products (38). Inactivation was validated with the TCID-50 assay.

### Cell culture and maintenance

The generation of THP-1 knockout cells (ATCC, TIB-202) has been described previously (40). THP-1 cells were maintained in Roswell Park Memorial Institute 1640 (RPMI) supplemented with 10% fetal bovine serum (FBS), minimum essential media (MEM) nonessential amino acids (1:100, Corning, cat# 25-025), 1% penicillin/streptomycin, sodium pyruvate (1 mM), and l-glutamine (2 mM) in a humidified 37°C, 5% CO_2_ incubator at a density of 5 × 10^5^ to 2 × 10^6^ cells/mL. For differentiation into macrophages, THP-1 cells were plated at a density of 2 × 10^5^ cells/well in a sterile U-bottom 96-well plate and differentiated overnight in RPMI, 2% FBS, penicillin/streptomycin, l-glutamine (2 mM), and phorbol 12-myristate 13-acetate (PMA) 5 ng/mL (41). Human lung carcinoma cell line A549 expressing human angiotensin-converting enzyme 2 with HA-FLAG (A549-ACE2) was obtained from BEI Resources, NIAID (NR-523522). A549-ACE2 cells were maintained in 10% FBS in Dulbecco’s modified Eagle medium (DMEM) with 1% penicillin/streptomycin, and l-glutamine (2 mM).

### Cytokine measurement

IL-18 was measured with the human IL-18 ELISA Kit (MBL, Woburn, MA), according to the manufacturer’s instructions, using cell culture supernatant at a 1:5 dilution. Data were acquired on a SpectraMax M2 with a lower limit of detection of 12 pg/mL. Additionally, cytokines from cell culture supernatants (IL-18, IL-1β, and TNF-α) were measured using a custom multiplex kit (U-plex) from Meso Scale Diagnostics (MSD, Rockville, MD), according to the manufacturer’s protocol and data were acquired on a MESO QuickPlex SQ 120.

### Viral inoculation of macrophages

Unless otherwise stated, all inoculation of THP-1 cells with SARS-CoV-2 was performed at a multiplicity of infection (MOI) of 0.2 for SARS-CoV-2. All *in vitro* data presented in graphic form represent data from at least in three independent experiments. Media were aspirated from cell culture wells and replaced with RPMI containing 2% heat-inactivated FBS (R2) and either SARS-CoV-2 or nigericin (5 µM) (MilliporeSigma, Burlington, MA) and LPS (1 µg/mL), or no additional reagents (mock/media control). Supernatant was collected for downstream assays at the timepoints indicated. For inhibiting ACE2-spike interaction, a neutralizing spike antibody was purchased from Sino Biological (cat# 40150-D001) and incubated with the virus for 1 h at 4°C prior to inoculation.

Peripheral blood mononuclear cells (PBMCs) from deidentified human blood leukopaks were isolated by Ficoll-Hypaque gradient centrifugation. Monocytes were isolated using the RosetteSep Immunodensity Cell Separation (Stem Cell Technologies cat# 15028) for human monocytes and primary monocytes were immediately used after isolation.

### Flow cytometry assessment of caspase-1 cleavage and ASC speck formation

For caspase-1 cleavage, a fluorescent caspase-1 inhibitor probe 660-YVAD-FMK, FLICA660, (ImmunoChemistry Technologies, cat# 9122) was added to supernatant at 24 h post-infection and incubated for 1 h at 37°C. Cells were washed, and other surface and intracellular staining proceeded. For ASC speck flow staining, after cells were fixed and permeabilized (BioLegend cat# 421403), ASC-PE antibodies were added at 0.5 µL/2e5 cells overnight at 4°C. Cells were washed with perm buffer after overnight incubation. ASC specks were gated on high ASC-H and a lower ASC-W compared to the lower ASC-H population (42).

### Measurement of LDH activity

Lactate dehydrogenase (LDH) activity as a measure of cell death was assayed in cell-free supernatants from cell culture using the Cytotoxicity Detection Kit (LDH) from Roche (cat# 11644793001) according to the manufacturer’s instructions.

### Infections of human nasal epithelial cultures

hNECs (Promocell) were grown to confluence in 24-well Falcon filter inserts (0.4 µm pore; 0.33 cm^2^; Becton Dickinson) using PneumaCult-Ex Plus Medium (Stemcell, cat #05041). Confluence was determined by a trans-epithelial electrical resistance (TEER) reading above 250 Ω. The cells were then differentiated at an air-liquid interface (ALI) for 3–4 weeks until the presence of ciliated cells was obvious across most of the wells, using ALI medium as basolateral medium as previously described (43). For infection, virus was added to the apical side in 2.5% FBS in DMEM at an MOI of 0.1 PFU/cell for 1 h at 33°C. After washing with Dulbecco’s phosphate-buffered saline (DPBS), the apical surfaces were kept dry until the indicated times. At the indicated times post-infection, 150 µL of infection media (IM) was added to the apical surface, incubated at 33°C for 15 min, and then collected. A single male donor, age 41 was used for the hNEC cultures in this paper.

### Exogenous cytokine treatment of hNECs

The hNEC cultures were incubated with either vehicle control (IM), recombinant IL-18 (50 ng/mL, R&D Systems, cat# B001-5), recombinant IL-1β (50 ng/mL, PEPROTECH, cat# AF-200-01B), both IL-18 and IL-1β, or recombinant universal type 1 interferon (1,000 U/mL, PBL Assay Science, cat# 11200-2) all diluted in basolateral media for 4 h at 37°C. The cells were then infected from the apical side with an MOI of 0.1 PFU/cell or mock-infected with IM at 37°C in 200 µL. After 1 h, the cells were washed with DPBS, and the apical surfaces were kept dry until the indicated times. Basolateral media with the indicated cytokines were maintained on the cells throughout the experiment. At the indicated number of hours post-infection (HPI), IM (150 µL) was added to the apical side and incubated for 15 min before collecting and storing at −80°C.

### Tissue culture infectious dose (TCID_50_) assay

Vero E6-TMPRSS2 cells were plated in 96-well plates and cultured until 90%–100% confluent. The cells were washed two times in DPBS, then covered with 180 µL of IM. Tenfold serial dilutions of virus at each time point were made in a separate plate. Each dilution was plated in replicates of four. Infection proceeded for 5 days at 37°C, the cells were fixed with 4% formaldehyde, and stained with naphthol blue-black solution. Viral-induced cytopathic effect was scored visually and the titer of infectious virus was calculated based on the Reed-Muench method (37, 44).

### RT-qPCR and qPCR

Supernatant samples were inactivated in 0.5% Triton X-100 before proceeding with viral RNA extraction (QIAamp viral RNA mini kit, cat# 52906). The RT-qPCR assay followed the U.S. CDC-designed research use only (RUO) RT-PCR assay kits (IDT, cat# 10006713) with TaqPath 1-step RT-qPCR masterMix (ThermoFisher, cat# A15299). Standard curves were included in all RT-qPCR assays (IDT, cat# 10006625), with a range of detection of 320 copies to 200,000 copies/reaction. The CDC-recommended cycling condition was used (45). The QIAGEN RNeasy plus kit (QIAGEN, cat# 74136) was used for RNA extraction. cDNA was synthesized using Superscript IV First-Strand Synthesis System kit (Thermofisher, cat# 18091050). Primers and probes for IL-1β along with PrimeTime qPCR assay master mix, were purchased from IDT.

### Immunofluorescence imaging

SARS-CoV-2-infected hNEC cultures were fixed by incubation with 4% paraformaldehyde for 24 h, then permeabilized with PBS containing 0.2% Triton X-100 for 10 min. The cells were subsequently incubated with anti-dsRNA (Sigma, MABE1134), anti-β tubulin antibody (Abcam, ab21057), anti-SARS-CoV-2 spike protein antibody (Sino Biological, 40150-D001), and anti-SARS-CoV-2 nucleocapsid protein antibody (Rockland, 200-401-A50) at 1:200 dilution for 1 h at room temperature (RT), followed by incubation with 1:500 diluted goat anti-mouse IgG AF555 and goat anti-rabbit AF488 (Invitrogen, A-21422 and A32731) for 1 h at RT. Nuclear stain (ThermoFisher, H3569) was then added prior to adding mounting media. The fluorescence images were recorded using a Zeiss LSM 900 microscope using a 20× objective lens.

### Statistical analysis

Unpaired Student’s *t* tests were used to calculate *P* values by comparing the means of two groups in all the cytokine plots. Wilcoxon rank-sum test was used to calculate *P* values for comparison between viral load quantities with and without cytokine pre-treatment at specific time points. The sample size, number of replicates, and the type of analysis performed are described in the figure legends where applicable. For all studies, significance is defined as *P* ≤ 0.05 (*P < 0.05; **P < 0.01; ***P < 0.001; ****P < 0.0001). Significance between all groups is not always depicted in figures. Statistical tests were performed in R (v4.3.2) and prism (10.1.1).

## RESULTS

### SARS-CoV-2 activates macrophage NLRP3 inflammasomes *in vitro*

**Fig 1:**
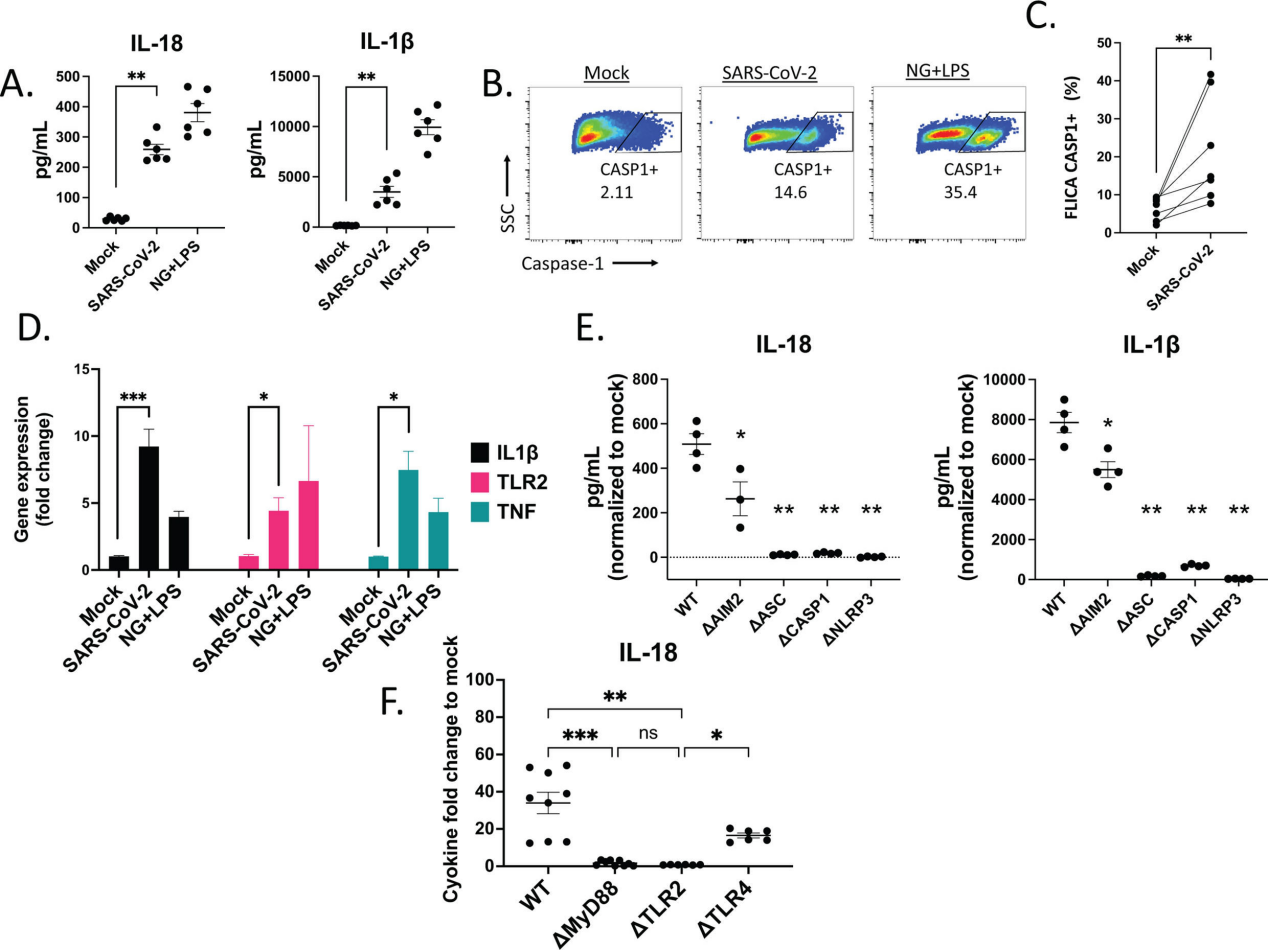
SARS-CoV-2 activates NLRP3 inflammasomes in macrophages *in vitro*. (**A**) PMA-stimulated THP-1 cells were inoculated with media alone (Mock), SARS-CoV-2 (MOI = 1), or nigericin and lipopolysaccharide (NG + LPS). Supernatants were collected 24 h post-inoculation (HPI) and IL-18 (left panel), IL-1β (right panel) were measured at 24 HPI by ELISA. Significance was tested using unpaired Mann-Whitney test compared with mock. (**B**) Representative gating of FLICA CASP1^+^ THP-1 cells and (**C**) pooled independent experiments after 24 h stimulation with mock or SARS-CoV-2. Number below gate represents % CASP1^+^ THP-1 cells. Significance was tested using paired Wilcoxon matched-pairs signed rank test. (**D**) Gene expression (fold change to mock) of IL1β, TLR2, and TNF 24 HPI. Significance was tested using unpaired Mann-Whitney test compared with mock. (**E**) THP-1 cells with the indicated inflammasome-related genes, AIM2, ASC, CASP-1, and NLRP3, disrupted via CRISPR-cas9 (Δ) were inoculated with SARS-CoV-2, NGLPS, or media control and supernatants were collected 24 HPI. IL-18 (Left) and IL-1β (Right) levels were measured by ELISA and normalized to mock condition. ΔHUMCYC cells are labeled as “WT.” Significance was tested using unpaired Mann-Whitney test compared with WT. (**F**) WT, ΔMyD88, ΔTLR2, and ΔTLR4 THP-1 cells were inoculated with SARS-CoV-2 and supernatants were collected 24-HPI. IL-18 was measured by ELISA and normalized to mock condition, and fold change to mock is shown. Significance was tested using unpaired Mann-Whitney test compared with the indicated conditions.

Data from our group and others (7, 10–15) strongly support a role for inflammasome activation in SARS-CoV-2 infection. Monocytic cells are one of the primary producers of inflammasome-associated cytokines in many viral infections, and evidence supports their role in activating inflammasomes in response to SARS-CoV-2 (10, 11, 15). To assess whether SARS-CoV-2 directly activates inflammasomes in macrophages, we inoculated macrophage-like THP-1 cells with SARS-CoV-2/Wu-1 virus, nigericin and LPS (NGLPS, a potent activator of the NLRP3 inflammasome [46]), or media alone. SARS-CoV-2 exposure for 24 h led to significantly increased amounts of both IL-18 and IL-1β in the supernatants compared to the media control (Fig. 1A), as well as increased cell death, as measured by LDH in the supernatant, which was comparable to the amount induced by NGLPS (Fig. S1A). Because release of mature IL-18 and IL-1β requires cleavage of pro-IL-18 and pro- IL-1β by activated caspase-1, we used a cleaved caspase-1 probe, FLICA660, to assess caspase-1 cleavage in THP-1 cells inoculated with SARS-CoV-2. The percentage of FLICA CASP-1 positive (CASP1^+^) cells was significantly higher in response to SARS-CoV-2 compared to media (Fig. 1B and C). To assess whether SARS-CoV-2 primes THP-1 cells, we examined pro-IL-1β, TLR2, and TNF gene expression 24 h post-inoculation. Pro-IL-1β, TLR2, and TNF expression all significantly increased upon SARS-CoV-2 inoculation (Fig. 1D). Next, we used a panel of CRISPR-Cas9-modified THP-1 cells, each containing a disruption of one of four inflammasome genes to define the cellular components required for inflammasome activation (40, 41). The ΔHUMCYC cell line (WT), which was derived from the same THP-1 cells and lacks a human pseudogene (HUMCYCPS3), was used to control for CRISPR-Cas9 off-target effects. As with the NGLPS control, SARS-CoV-2 inoculation of cells lacking NLRP3, ASC, or CASP1 did not lead to inflammasome activation at 24 h post-inoculation, as measured by IL-18 and IL-1β production (Fig. 1E). We hypothesized that AIM2, a dsDNA inflammasome sensor, is not required for the inflammasome response to SARS-CoV-2, an RNA virus. Consistent with this, cells lacking AIM2 (ΔAIM2) produced IL-1β and IL-18 in response to SARS-CoV-2, although not as robustly as wild-type (WT) THP-1 cells. Disruption of NLRP3 completely negated IL-18 and IL-1β production in response to SARS-CoV-2, suggesting NLRP3, but not AIM2, is a required sensor for inflammasome activation and cytokine production in macrophages. In contrast, deficiency of NLRP3, ASC, or CASP1 reduced LDH activity compared to WT THP-1 cells inoculated with SARS-CoV-2 but not to levels observed with the media control (Fig. S1B). These data suggest that while inflammasome-mediated pyroptosis is responsible for some SARS-CoV-2-induced cell death, other cell death pathways may also be activated (47). Next, to investigate the pattern recognition receptor responsible for NLRP3 inflammasome activation, we inoculated ΔMyD88, ΔTLR2, and ΔTLR4 THP-1 cells with SARS-CoV-2. Cytokine production and IL-1β gene expression revealed that the inflammasome activation requires sensing by TLR2 and signaling by its downstream adaptor, MyD88, for signaling (Fig. 1F; Fig. S1C). We ruled out several individual structural SARS-CoV-2 proteins as the dominant ligand of TLR2 by adding them to THP-1 cells and measuring IL-18 and IL-1β production (Fig. S1D). These data suggest that the intact virion with viral RNA is required to prime the inflammasome, with isolated viral proteins insufficient. These results demonstrated that SARS-CoV-2 directly primes inflammasomes via the TLR2-MyD88 pathway and activates canonical NLRP3 inflammasome in a human macrophage cell line.

### NLRP3 inflammasome activation is independent of productive replication, receptor-mediated entry, and actin-dependent uptake of SARS-CoV-2

**Fig 2:**
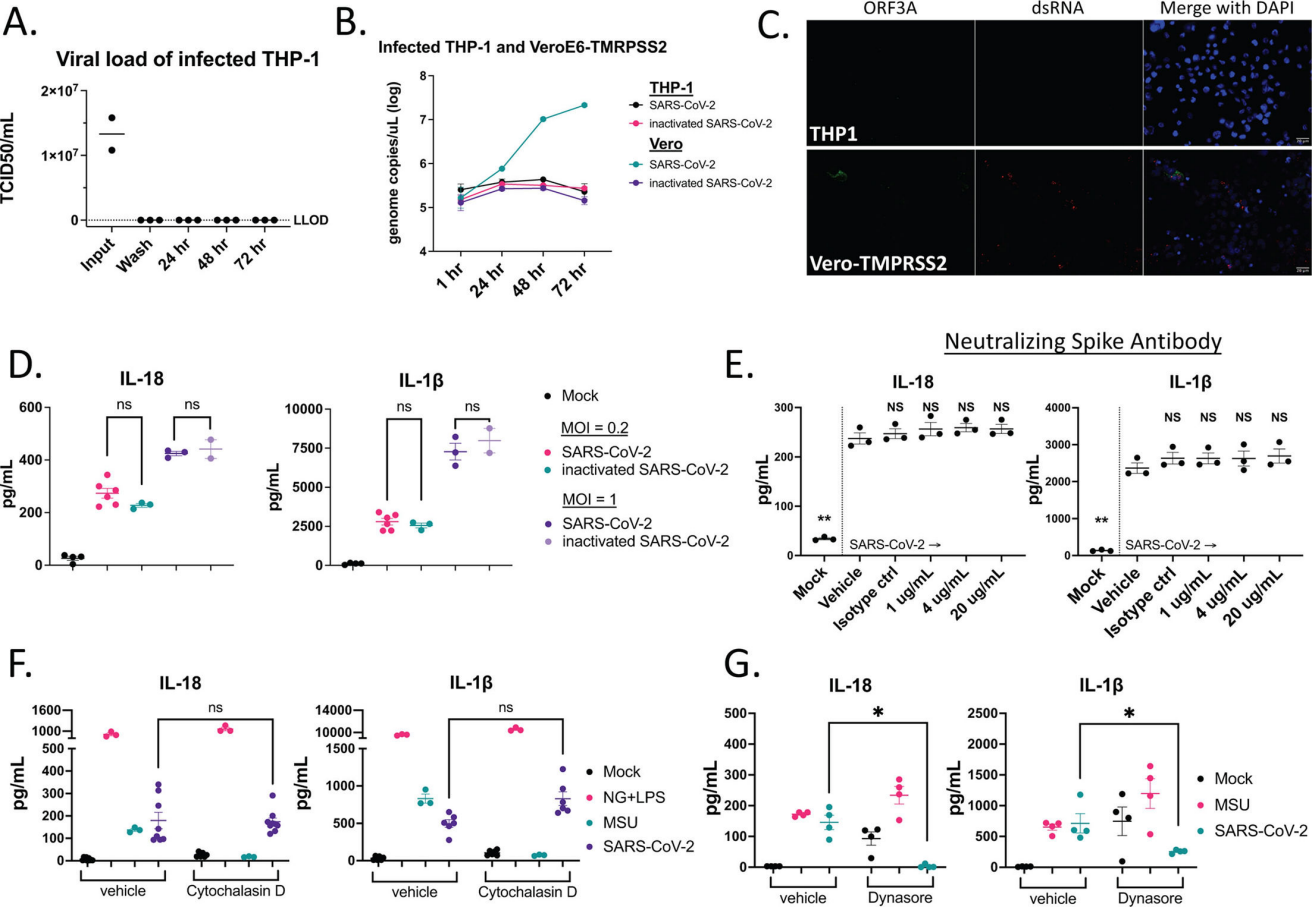
NLRP3 inflammasome activation is independent of productive replication, receptor-mediated entry, and actin-dependent uptake of SARS-CoV-2. (**A**) THP-1 cells were inoculated with SARS-CoV-2 at MOI of 1 then washed three times with PBS. Last wash after inoculation (Wash) and culture supernatants are collected at indicated time points for measurement of infectious virus by TCID_50_ assay. (**B**) THP-1 cells and VeroE6-TMPRSS2 cells were inoculated with SARS-CoV-2 and β-propiolactone treated SARS-CoV-2 (inactivated-SARS-CoV-2) at MOI of 0.2 and viral load is measured at the indicated HPI. (**C**) Immunofluorescent staining of SARS-CoV-2 nonstructural protein ORF3a (green), dsRNA (red), and nucleus (blue) in THP-1 cells and VeroE6-TMPRSS2 cells infected with SARS-CoV-2. Scale bars, 20 µm. (**D**) THP-1 cells and VeroE6-TMPRSS2 cells were inoculated with SARS-CoV-2 and inactivated-SARS-CoV-2 at MOI of 0.2 and 1. Supernatants were collected at 24 HPI, and IL-18 (left panel) and IL-1β (right panel) were measured by ELISA. (**E**) THP-1 cells inoculated with SARS-CoV-2 pretreated with neutralizing spike antibody at 1, 4, 20, and 100 µg/mL. Supernatants were collected at 24 HPI, and IL-18 (left panel) and IL-1β (right panel) were measured by ELISA. (**F**) THP-1 cells inoculated with mock, SARS-CoV-2, NG + LPS, or monosodium urate crystal (MSU) pretreated with cytochalasin D for 15 min. Supernatants were collected at 24 HPI, and IL-18 (left panel) and IL-1β (right panel) were measured. (**G**) THP-1 cells inoculated with mock, SARS-CoV-2, or monosodium urate crystal (MSU) pretreated with Dynasore for 15 min. Supernatants were collected at 24 HPI and IL-18 (left panel), IL-1β (right panel) were measured. Statistical tests in Fig. 2 were all using unpaired Mann-Whitney test compared with the indicated conditions.

SARS-CoV-2 tropism for macrophages is not fully understood. We confirmed that THP-1 and primary human monocytes express the canonical SARS-CoV-2 receptor, ACE2, at a lower level compared to VeroE6-TMPRSS2 cells (Fig. S2A). To test if replication is required to activate inflammasomes, we inoculated THP-1 cells with SARS-CoV-2 and measured viral load by TCID_50_. Infectious virus was not detected from supernatants 24–72 HPI (Fig. 2A). We compared SARS-CoV-2 genome copies at 1, 24, 48, and 72 HPI of VeroE6-TMPRSS2 and of THP-1 cells. In contrast to results in VeroE6, genome copies were not significantly different between β-propiolactone-inactivated SARS-CoV-2 genome copies and live virus inoculated THP-1 or between 1 and 72 h, suggesting no productive replication in THP-1 cells (Fig. 2B). In addition, double-stranded RNA (dsRNA; a marker of viral RNA replication complex formation) and the SARS-CoV-2 nonstructural protein, ORF3a, were detected by immunofluorescence imaging in VeroE6-TPMRSS2, but not in THP-1 cells (Fig. 2C). β-Propiolactone-inactivated SARS-CoV-2 induced similar level of IL-18 and IL-1β at MOI of 0.2 and 1 in THP-1s (Fig. 2D). Taken together, these data suggest that viral replication is not required for inflammasome activation in THP-1 cells.

To determine if inflammasome activation was dependent on receptor-mediated entry, we incubated SARS-CoV-2 with neutralizing spike-specific antibodies before adding the mixture to THP-1 cells. IL-18 and IL-1β production was induced over a range of antibody concentrations and to levels similar to those seen with virus alone (Fig. 2E; Fig. S2B). Another common entry mechanism by viruses is endocytosis, which can be grouped into different types based on the nature of the cargo and the factors involved (48). We treated THP-1 cells with cytochalasin D, a mycotoxin that inhibits actin polymerization and actin-dependent endocytosis (Fig. S2C). Treatment with cytochalasin D did not reduce IL-18 and IL-1β production in response to SARS-CoV-2 but reduced inflammasome activation by monosodium urate crystals (MSU), a PAMP capable of inducing the NLRP3 inflammasome (Fig. 2F). To further investigate, we used a dynamin inhibitor, Dynasore. Dynamin is necessary for clathrin-mediated endocytosis. Inhibition of dynamin with Dynasore reduced SARS-CoV-2 induction of IL-18 and IL-1β, but not MSU-mediated inflammasome activation (Fig. 2G). This suggests that the entry of SARS-CoV-2 into THP-1 cells is mediated through a dynamin-dependent process, most likely clathrin-mediated endocytosis, which is independent of the actin polymerization used in the uptake of MSU crystals. Taken together, these results indicate that SARS-CoV-2 can induce inflammasome activation in macrophages independent of virus receptor-mediated entry and actin-dependent processes but requires dynamin-dependent endocytosis.

### SARS-CoV-2 does not induce inflammasome activation in hNEC cultures and exogenous IL-18 and IL-1β do not inhibit SARS-CoV-2 replication

**Fig 3:**
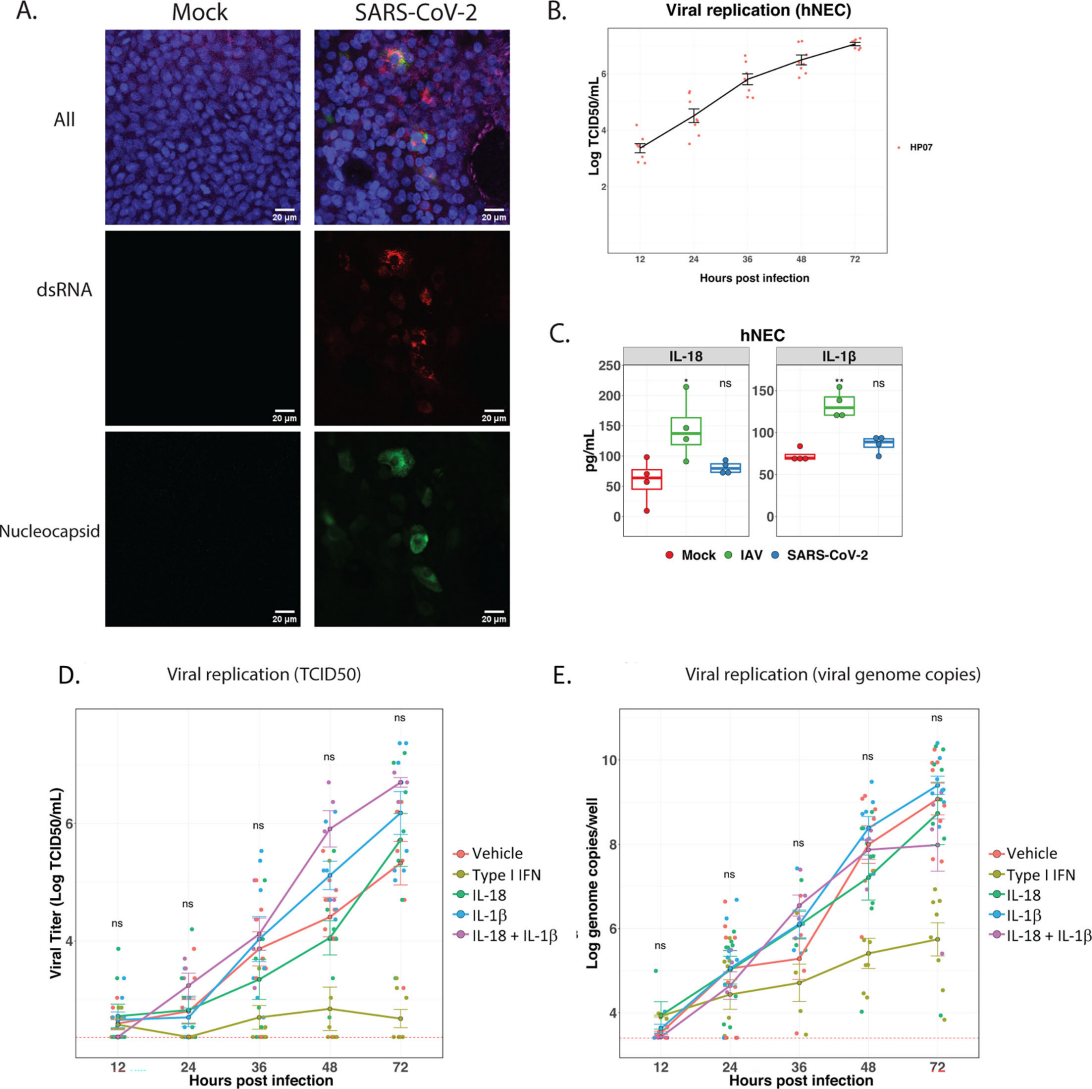
In SARS-CoV-2-infected human nasal epithelial cell (hNEC) cultures, neither IL-18 nor IL-1β were produced and exogenous IL-18 and IL-1β did not inhibit viral replication. (**A**) Primary hNECs were cultured in air-liquid interface transwells, inoculated with media alone (left panels) or SARS-CoV-2 at MOI of 0.1 (right panels) for 48 h. Cells were stained with beta-4 tubulin (purple, top merged), nucleus marker DAPI (blue, top merged), dsRNA (red, top merged, and middle), and SARS-CoV-2 nucleocapsid protein (green, top merged, and bottom). Scale bars, 20 µm. (**B**) SARS-CoV-2 TCID_50_ from apical side at 12, 24, 36, 48, and 72 h post-infection (HPI). (**C**) IL-18 (left panel) and IL-1β (right panel) levels of basolateral supernatant at 48 HPI were measured by MSD. Each dot represents an individual well performed in one experiment from one hNEC culture. (**D and E**) Cytokines were added to basolateral media, respectively, as indicated in the diagram. Supernatants from the apical side were collected at various time points shown in the diagram. hNECS were incubated with media (orange), type I Interferon (yellow-green), IL-1β (blue), IL-18 (green), or IL-18 and IL-1β (pink) in the basolateral media for 4 h prior to infection with SARS-CoV-2. Inoculum was added to the apical surface of hNEC culture for 1 h. Apical virus was quantified by (**D**) RT-qPCR and (**E**) TCID_50_ at 12, 24, 36, 48, and 72 HPI. Each dot represents an individual well performed in a total of two experiments from two hNEC cultures. Differences between conditions containing vehicle and type I interferon, vehicle, and IL-18 with IL-1β, vehicle and IL-18, vehicle and IL-1β were determined by a Wilcoxon rank-sum test. NS indicates *P* values >0.05. None of the above comparisons were significant except for vehicle and type I Interferon at 48 and 72 HPI; therefore, only NS is shown above each time point. Experiment was performed two times as shown.

Given that we found inflammasome activation in macrophages was independent of viral replication, we sought to determine if inflammasomes were activated in cells that are known to be permissive for replication. Consistent with other reports, we found that SARS-CoV-2 replicates in primary, differentiated hNEC cultures, as demonstrated by the detection of viral antigen (nucleocapsid, N) and of dsRNA by immunofluorescence microscopy (Fig. 3A) and by infectious virus production (Fig. 3B) (49, 50). The inflammasome is activated in airway epithelial cells by viruses, such as rhinovirus and IAV (51, 52). To determine if SARS-CoV-2 infection of hNEC cultures results in inflammasome activation, we measured inflammasome-associated cytokines in the basolateral cell culture supernatants from SARS-CoV-2-infected hNEC cultures compared to IAV- or mock-infected hNEC cultures. Unlike in THP-1 cells exposed to SARS-CoV-2, we did not detect increased levels of IL-18 and IL-1β in SARS-CoV-2-infected hNEC cultures at 12, 24 and 48 HPI compared to negative controls (Fig. 3C; Fig. S3A). The levels of both inflammasome cytokines measured remained at baseline at all time points, indicating that SARS-CoV-2 infection of hNEC cultures does not result in IL-18 or IL-1β production despite robust viral replication. In contrast, IL-18 and IL-1β were produced 48 h after IAV infection of hNEC, confirming that hNEC cultures can activate the inflammasome in response to RNA viruses. These findings are consistent with our prior studies demonstrating that while hNECs are susceptible and permissive for both IAV and SARS-CoV-2, the cellular and innate responses are markedly different (53–55).

Because circulating IL-18 and IL-1β are elevated in severe COVID-19 (7, 10), and SARS-CoV-2 exposure leads to the release of these inflammasome-associated cytokines in macrophages, the inflammasome may be a therapeutic target during the inflammatory phase of the disease. However, if these cytokines directly inhibit SARS-CoV-2 replication in epithelial cells, inhibiting inflammasome activation could worsen disease severity or lead to prolonged viral shedding. To determine whether these inflammasome-induced cytokines inhibit SARS-CoV-2 replication, we infected hNEC cultures with SARS-CoV-2 in the presence of media alone, type I IFN, IL-18, and/or IL-1β and measured infectious virus production and viral genome copies at 24, 48, and 72 HPI (Fig. S3B). Type I IFN is known to suppress SARS-CoV-2 replication (56). While type I IFN significantly reduced SARS-CoV-2 replication at 48 and 72 HPI, neither IL-18 or IL-1β alone nor the combination of the two suppressed viral replication at any time point as determined by either TCID_50_ or RT-qPCR assay (Fig. 3D and E). In addition to hNEC cultures, we also examined cytokine effects on SARS-CoV-2 replication in A549 cells, a human alveolar basal epithelial cell line, transduced with a lentivirus encoding human ACE2 (A549-hACE2) (57). In agreement with our hNEC culture data, neither IL-18 or IL-1β alone, nor the combination, suppressed viral replication in A549-hACE2 cells (Fig. S3C and D). These data suggest the inflammasome-produced cytokines IL-18 and IL-1β do not directly inhibit viral replication in hNEC cultures or A549-ACE2 cells. Taken together, SARS-CoV-2 does not induce inflammasome activation in infected nasal epithelial cells and the inflammatory cytokines do not play a direct antiviral role in infected nasal epithelial cells.

### Primary monocytes activate inflammasomes and upregulate macrophage marker CD206 in response to SARS-CoV-2

**Fig 4:**
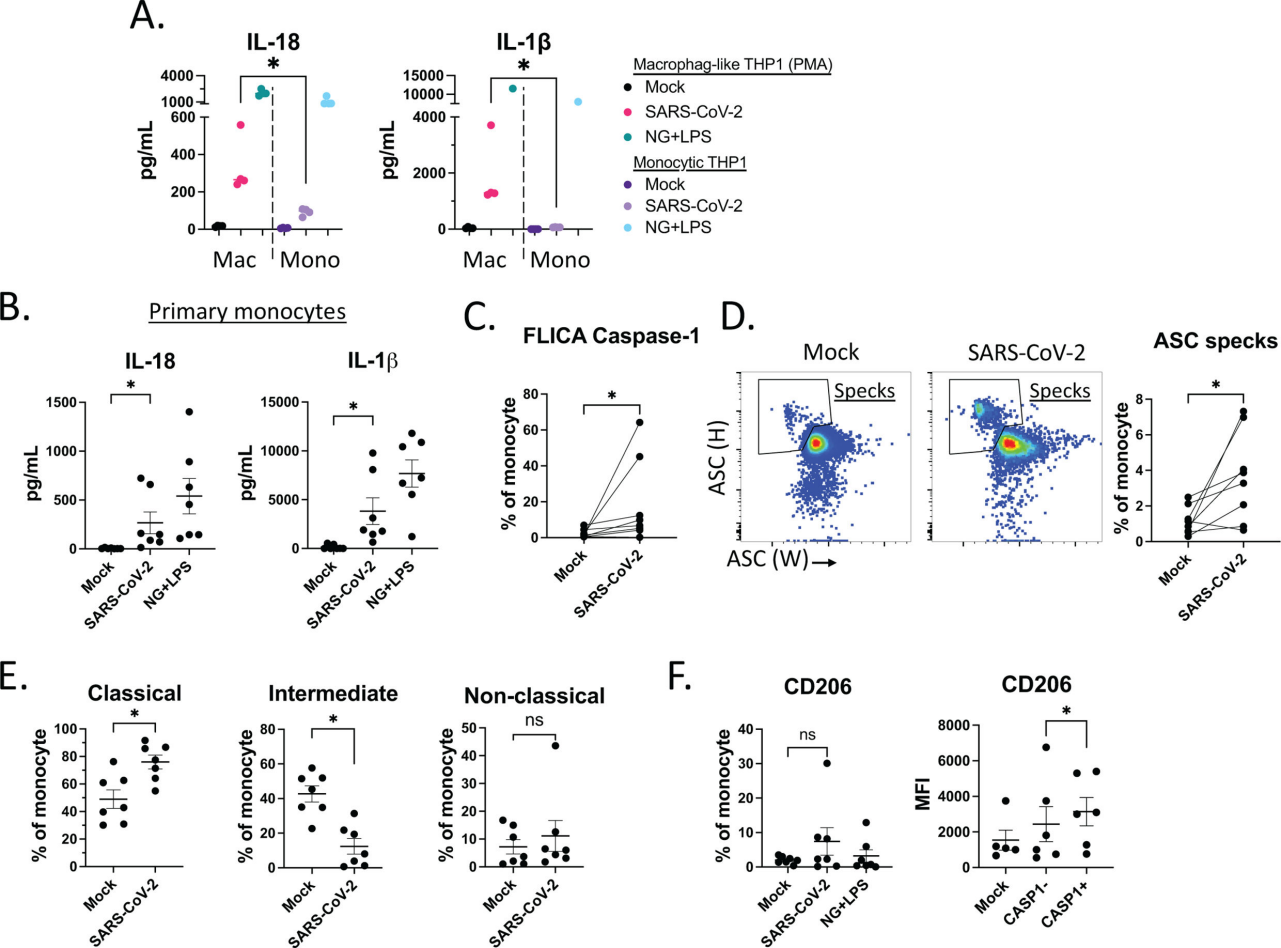
Primary monocytes activate inflammasomes and upregulate macrophage marker CD206 in response to SARS-CoV-2. (**A**)THP-1 cells treated with PMA (macrophage-like THP1) or without PMA (monocytic THP-1) were inoculated with mock, SARS-CoV-2, or NG + LPS. Supernatants were collected 24 HPI, and IL-18 (left panel) and IL-1β (right panel) were measured. (**B**) Primary human monocytes were stimulated with media, SARS-CoV-2, or NG + LPS. Supernatants were collected 24 HPI, and IL-18 (left panel) and IL-1β (right panel), and (**C**) CASP1^+^ population were measured. (**D**) Representative gating (left two) and independent experiments (right) of ASC speck^+^ primary monocytes after 24 HPI with mock or SARS-CoV-2. (**E**) Percentages of classical (CD14^+^CD16^neg^), intermediate (CD14^+^CD16^+^) and non-classical (CD14^dim^CD16^+^) monocytes after stimulation with media, SARS-CoV-2, or NG + LPS. (**F**) Percentages of CD206^+^ monocytes after stimulation with media, SARS-CoV-2, or NG + LPS (left). CD206 MFI of mock-treated monocyte, SARS-CoV-2 treated CASP1-, or SARS-COV-2-treated CASP1^+^ monocytes (right). Each dot represents a unique healthy donor. Figure 4A significance was tested using unpaired Mann-Whitney test compared with the indicated conditions. All other figures used paired Wilcoxon matched-pairs signed rank test.

Monocytes are found in the bloodstream and can migrate into tissues and differentiate into macrophages. We hypothesized that THP-1 cells could sense SARS-CoV-2 and activate inflammasomes without differentiation into their macrophage phenotype. Surprisingly, monocyte-like THP-1 cells in suspension culture without PMA treatment produced significantly less IL-18 and IL-1β in response to SARS-CoV-2 exposure (Fig. 4A). This suggests that monocytes have a reduced capacity to activate inflammasomes in response to SARS-CoV-2 when compared to macrophages. Next, we inoculated primary human monocytes from healthy donors with SARS-CoV-2. Both IL-18 and IL-1β were induced 24 HPI, but at low levels (Fig. 4B), confirming that primary monocytes can activate inflammasomes in response to SARS-CoV-2. There was a significant increase in CASP1^+^ monocytes, indicating caspase-1 cleavage (Fig. 4C), as well as significant increases in the ASC speck population, in response to SARS-CoV-2 (Fig. 4D). Inoculation with SARS-CoV-2 resulted in no change in frequency of non-classical monocytes (CD14dimCD16+), increased classical monocytes (CD14^+^CD16^−^) and decreased intermediate monocytes (CD14^+^CD16^+^) (Fig. 4E). To explore whether monocytes undergo phenotypic changes upon activation by SARS-CoV-2, we measured levels of CD206, a macrophage differentiation marker. The percentage of CD206^+^ monocytes was slightly increased in the SARS-CoV-2-stimulated monocyte population. Interestingly, CD206 levels were significantly higher in CASP1^+^ monocytes compared to CASP1^−^ monocytes. The increased CD206 level, but not the CD206^+^ population, suggests that CD206^+^ upregulation drives CD206^+^ monocytes towards a more phagocytic phenotype, which may contribute to sensing SARS-CoV-2 and inflammasome signaling (58) (Fig. 4F). Consistent with our THP-1 data, these data suggest that macrophages, but not monocytes, are the primary activators of inflammasomes in response to SARS-CoV-2. Taken together, these findings demonstrate that primary monocytes induce IL-18 and IL-1β, caspase-1 cleavage, and ASC specks, and begin to differentiate into macrophages upon interaction with SARS-CoV-2.

### Monocytic cells in the lungs of patients with COVID-19 cleave caspase-1, increase CD11b expression, and produce reactive oxygen species

**Fig 5:**
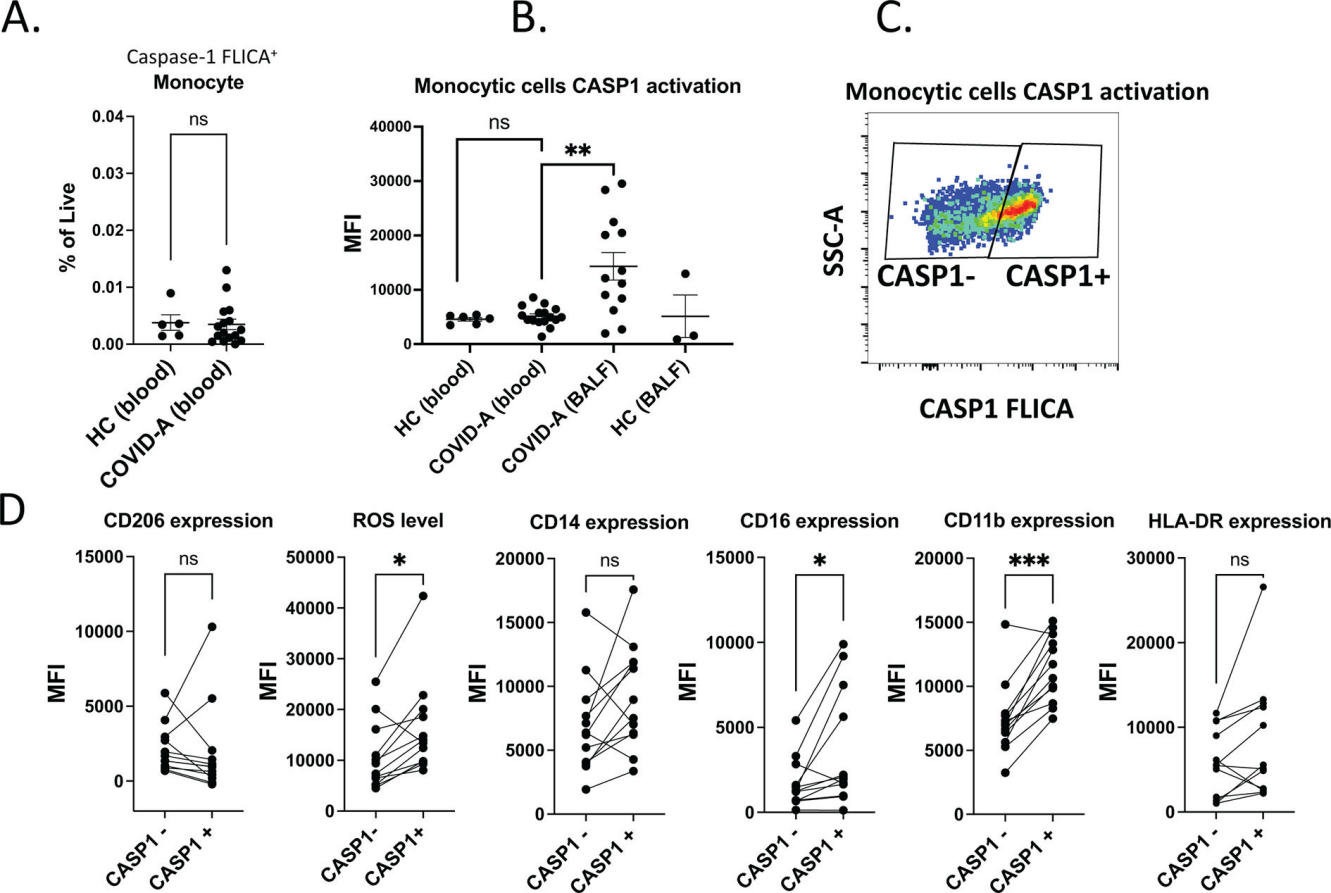
Monocytes from lung, but not periphery demonstrate evidence of inflammasome activation and markers of macrophage differentiation in patients with COVID-19. (**A**) % of CASP1^+^ monocytes in peripheral blood of healthy donors (HC) and patients with acute COVID-19 (COVID-A). Significance was tested using unpaired Mann-Whitney test compared with the indicated conditions. (**B**) FLICA CASP1 MFI of monocytic cells (CD14^dim/+^) from peripheral blood or BAL fluid of HC and COVID-A. Significance was tested using unpaired Mann-Whitney test compared with the indicated conditions. (**C**) Representative gating of CASP1^+^ monocytic cells from BAL fluid of COVID-A subject. (**D**) CD206, ROS, CD14, CD16, CD11b, and HLA-DR MFI of CASP1^−^ and CASP1^+^ monocytic cells in the BAL fluid of COVID-A subjects. Significance was tested using paired Wilcoxon matched-pairs signed rank test.

To assess inflammasome activation of monocytes and macrophages in patients with COVID-19,

we compared PBMC samples from patients hospitalized with acute COVID-19 (*n* = 16) to healthy donors (*n* = 3) (Table S1). Monocytes (HLADR^+^CD14^+^/low CD16^−/+^) showed little to no CASP1^+^ signal in both COVID-19 and healthy donors (Fig. 5A; Fig. S4A). Further, assessment of other immune cell types showed that circulating neutrophils were the only population with activated caspase-1 in blood (Fig. S4B). Direct sensing of SARS-CoV-2 by monocytes in the blood is unlikely because SARS-CoV-2 viremia is rare and isolation of infectious virus from blood has rarely been reported (59). To assess the tissue macrophage response to SARS-CoV-2, we investigated immune cells in bronchoalveolar lavage (BAL) fluid samples from patients hospitalized with acute COVID-19 (*n* = 9 with four patients with two time points) and healthy donors (*n* = 3) (Table S2). Compared to percentages of CASP1^+^ blood monocytes, BAL-derived monocytes/macrophages (HLADR^+^CD14^+^/low CD16^−/+^) showed increased FLICA CASP1 signal from the patients with COVID-19 (Fig. 5B). CD206 positive cells are alveolar macrophages, the most abundant myeloid cell type in BAL fluid (60). Next, we designated CD206 high monocytic cells as alveolar macrophages and CD206^low/−^ population as interstitial macrophages or monocytes (Fig. S5A) (61–64). We found that although monocytic cell frequencies are comparable between healthy donors and patients with COVID-19, the alveolar macrophage proportion was higher in healthy donors (Fig. S5B and C). Notably, the FLICA CASP1 signal on monocytes and alveolar macrophages was not significantly different and the CASP1^+^ monocytic cells did not express higher CD206 (Fig. 5C and D; Fig. S5D). Furthermore, CASP1^+^ monocytic cells showed elevated levels of reactive oxygen species (ROS) measured by dihydrorhodamine 123. CASP1^+^ monocytes also displayed higher expression of CD16 and CD11b (Fig. 5D). ROS production by macrophages and monocytes has been shown to contribute to sustained pulmonary infiltration of monocytes and monocyte-derived macrophages (65, 66). CD11b is an integrin known to mediate macrophage adhesion, migration, chemotaxis, and accumulation during inflammation (67–69). These data suggest that caspase-1 activation of airway monocytic cells, primarily macrophages, is associated with the production of ROS and a proinflammatory airway compartment.

## DISCUSSION

Our results confirmed NLRP3 as the intracellular sensor responsible for macrophage inflammasome activation by SARS-CoV-2 and demonstrated that productive replication of the virus is not required for inflammasome activation. We showed that direct inflammasome activation in response to SARS-CoV-2 requires ASC, CASP-1, and NLRP3. While SARS-CoV-2 exposure led to diminished cytokine production in AIM2-deficient THP-1 cells, AIM2 is neither necessary nor sufficient for SARS-CoV-2 inflammasome activation in macrophages. AIM2 has been shown to act downstream of NLRP3 inflammasome activation and can amplify the response to NLRP3 stimuli (70, 71), a possible explanation for the decrease in, but not loss of, inflammasome cytokines with disruption of AIM2 that we observed. A recent study found AIM2 inflammasome activation in monocytes from patients with COVID-19 (15). Our data support the idea that AIM2 inflammasome activation may take place after SARS-CoV-2 sensing by macrophages, but NLRP3 is the predominant inflammasome activated in response to direct SARS-CoV-2 inoculation. We extend prior work demonstrating a role for NLRP3 inflammasome activation in myeloid cells (11, 14, 15) by demonstrating the critical dependence on TLR2-MyD88 signaling as a priming step for SARS-CoV-2-induced inflammasome activation. Activation of TLR2 signaling may have important clinical consequences, given that a genome-wide association study identified an association between COVID-19-associated mortality and a polymorphism in TIRAP, a signal adaptor of TLR2 (72). Further studies investigating whether TLR2 polymorphisms in the general population correlate with COVID-19 disease severity may contribute to an improved understanding of the molecular determinants of COVID-19 outcomes.

Importantly, SARS-CoV-2-associated NLRP3 inflammasome activation is not dependent on productive viral replication in myeloid cells. Instead, it is likely macrophages sense virus and are activated as bystanders at the site of infection (14). This is consistent with recent work that found that SARS-CoV-2 only abortively infects monocytes/macrophages and may explain why treatment with monoclonal antibodies in the inflammatory phase of disease fails to reduce pathology (12, 15, 73). Conversely, we found that robust infection of replication-permissive hNEC cultures did not result in the production of the inflammasome-mediated cytokines IL-1β or IL-18, and that production of these cytokines during COVID-19 is likely due to myeloid cell sensing of SARS-CoV-2 rather than infection of respiratory epithelial cells. However, our experiments were limited to lower MOIs as more physiologic and 72 HPI, previously shown to be sufficient for peak viral replication to preserve hNEC culture architecture over the course of the experiments (53, 54). We also demonstrated that macrophage sensing of SARS-CoV-2 is independent of receptor-mediated entry and actin-dependent endocytosis.

There has been interest in therapeutic strategies targeting NLRP3 and/or IL-1β and IL-18 for COVID-19 (74, 75). IL-1β can have both direct and indirect antiviral effects in the context of other viral infections, but is also associated with a variety of pathological inflammatory disorders, including infections, autoimmunity, cardiovascular disease, and cancer (76–79). However, it is still unclear if inflammasome activation enhances immunopathology, or if elevated IL-1β and IL-18 are an appropriate response to uncontrolled viral replication during SARS-CoV-2 infection. Severe COVID-19 cases are associated with both higher viral levels and elevations in these and other proinflammatory cytokines (7). Therefore, it is possible that excessive inflammasome activation is a direct response to uncontrolled viral replication. IL-18 can mediate antiviral effects, as demonstrated with hepatitis B virus (80, 81) and vaccinia virus (82). While IL-18 contributes to the antiviral response in influenza through the activation of lymphocytes (83, 84), it does not appear to have a direct antiviral effect on rhinovirus (85). Similarly, IL-1β has been demonstrated to directly induce antiviral gene expression and restrict viral replication of vesicular stomatitis virus and hepatitis C virus (86, 87). Conversely, our results are consistent with the data in rhinovirus infection, where IL-18 and IL-1β had no direct effect on viral replication because these cytokines had no direct antiviral activity against SARS-CoV-2 in primary differentiated hNEC cultures or in an immortalized lung epithelial cell line (A549). These data support the use of anti-IL-1β and -IL-18 therapies in COVID-19.

Our system did not test indirect the effects of inflammasome-dependent cytokines on viral replication, such as cytokine elaboration from lymphocytes and dendritic cells, which is a limitation of the study. For example, IL-18 has been shown to prevent further tissue damage and loss of tissue function when acting upon Treg cells during influenza infection (88). Our system was limited to the human nasal epithelium, which lacks T cells. Therefore, we cannot rule out the possibility of a beneficial role of IL-18 in inflammatory resolution. While our study was limited to the assessment of antiviral effects in nasal epithelial cells and a lung adenocarcinoma cell line, clinical studies demonstrating a therapeutic benefit to non-specific immunosuppression with dexamethasone or specific inhibition of IL-1β and IL-1α with the IL-1 receptor antagonist anakinra support our data, suggesting a predominantly pathological rather than antiviral role for these cytokines (89–91). A study demonstrated differential response of the bronchial and nasopharyngeal airways to SARS-CoV-2 infection (92); therefore, it is possible that bronchial epithelial cells and small airway epithelial cells exhibit distinct responses to exogenous IL-18 and IL-1β or that SARS-CoV-2 induces activation of inflammasomes in these cells. However, we chose to study nasal epithelium because it is the portal of entry for SARS-CoV-2 and plays a critical role in suppressing viral infection (50, 93).

Monocytes in circulation have been reported to activate the inflammasome during COVID-19 (11, 13, 15). Our data indicate that circulating monocytes from COVID-19 patients do not demonstrate inflammasome activation directly *ex-vivo*. However, *in vitro* stimulation by SARS-CoV-2 showed that in response to SARS-CoV2 inoculation, primary monocytes do undergo caspase-1 cleavage and subsequent production of IL-18 and IL-1β. This is likely due to lack of viral sensing in the peripheral blood where the number of virions is magnitudes lower compared to that in the upper respiratory track, if virus is present at all (94). This discrepancy may also result from pyroptotic monocytes being excluded in our live-dead stains. The fact that caspase-1 cleaved primary monocytes upregulated CD206 *in vitro* suggest that inflammasome activation by SARS-CoV-2 occurs in distal airways and alveoli rather than in the periphery. Further experiments in relevant animal models are needed to tease apart whether factor(s) driving the difference in inflammasome activation in different tissue compartments exists.

In conclusion, our study demonstrates that SARS-CoV-2 can directly induce NLRP3 inflammasome activation via TLR2 priming in macrophages in the absence of productive viral replication. Conversely, the virus can productively infect primary differentiated human nasal epithelial cells without inflammasome activation. Our data indicate that IL-18 and IL-1β do not directly suppress SARS-CoV-2 replication in nasal epithelial cells. Activated macrophages in the airways become inflammatory and display oxidative bursts. These findings advance our understanding of innate sensing of SARS-CoV-2 and highlight areas for potential pharmacological intervention to improve COVID-19 treatment outcomes.

## Data Availability

The data that support the findings of this study are available through SeroNet, ImmPort database: https://www.immport.org/shared/study/SDY2753. For any additional information or requests for data, please contact Leon Hsieh at lhsieh6@jhmi.edu or Andrea Cox at acox@jhmi.edu

## SUPPLEMENTAL MATERIAL

Fig. S1 mbio.00810-24-s0001.pdf LDH level, IL1B gene expression, and cytokine levels in THP-1 cells in response to SARS-COV-2 or recombinant structural proteins.

Fig. S2 mbio.00810-24-s0002.pdf Evaluation of cellular ACE-2 level, neutralizing spike antibody activity, and cytochalasin D activity in the indicated cell lines.

Fig. S3 mbio.00810-24-s0003.pdf SARS-CoV-2 viral load in A549-ACE2 cells with exogenous IL-18 and IL-1β treatment.

Fig. S4 mbio.00810-24-s0004.pdf PBMC gating strategy and PBMC population in healthy donors and COVID-19 patients.

Fig. S5 mbio.00810-24-s0005.pdf BAL sample gating strategy and monocyte population in COVID-19 patients.

Supplemental legends mbio.00810-24-s0006.docx Legends for Fig. S1 to S5.

Table S1 mbio.00810-24-s0007.docx Demographics of subjects studied.

ASM does not own the copyrights to Supplemental Material that may be linked to, or accessed through, an article. The authors have granted ASM a non-exclusive, world-wide license to publish the Supplemental Material files. Please contact the corresponding author directly for reuse.
