# Supplementary figures and images for: Bystander monocytic cells drive infection-independent NLRP3 inflammasome response to SARS-CoV-2
Source: mBio. 2024 Sep 6;15(10):e00810-24. doi: 10.1128/mbio.00810-24 (PMC11481483; doi:10.1128/mbio.00810-24)

Supplemental Fig 1

A.

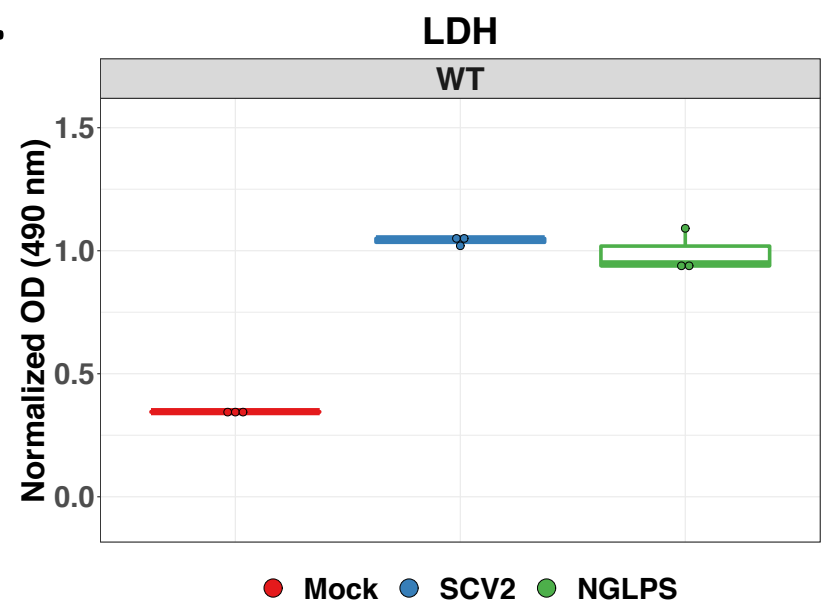

B.

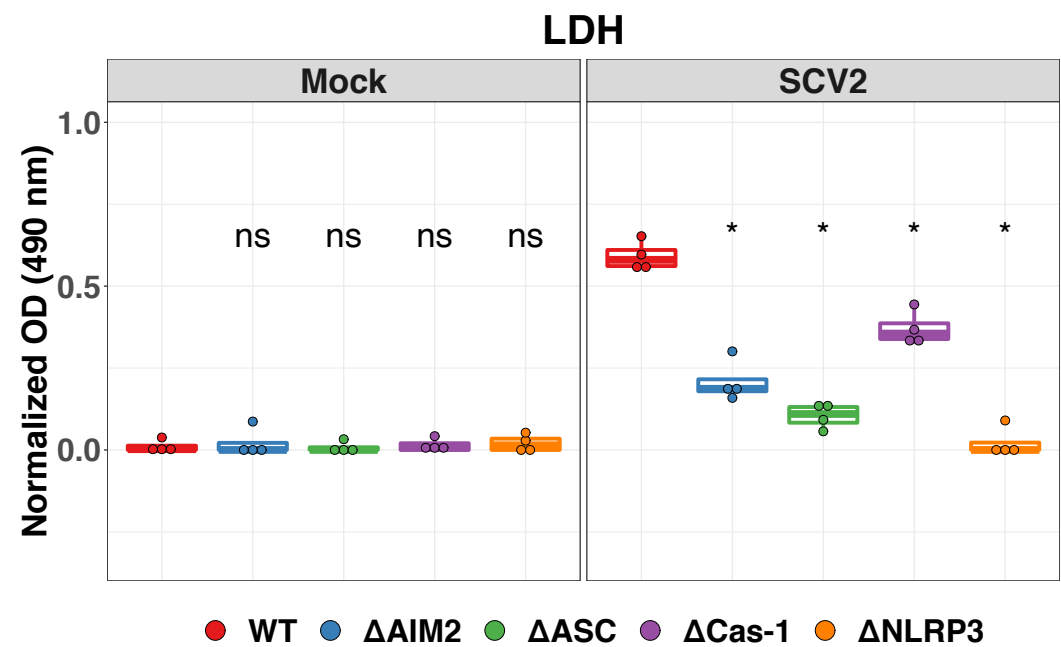

C.

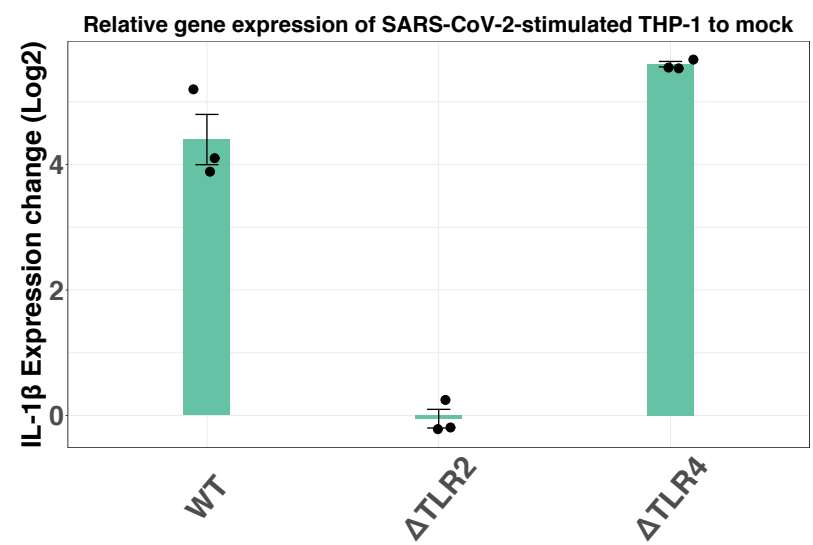

D.

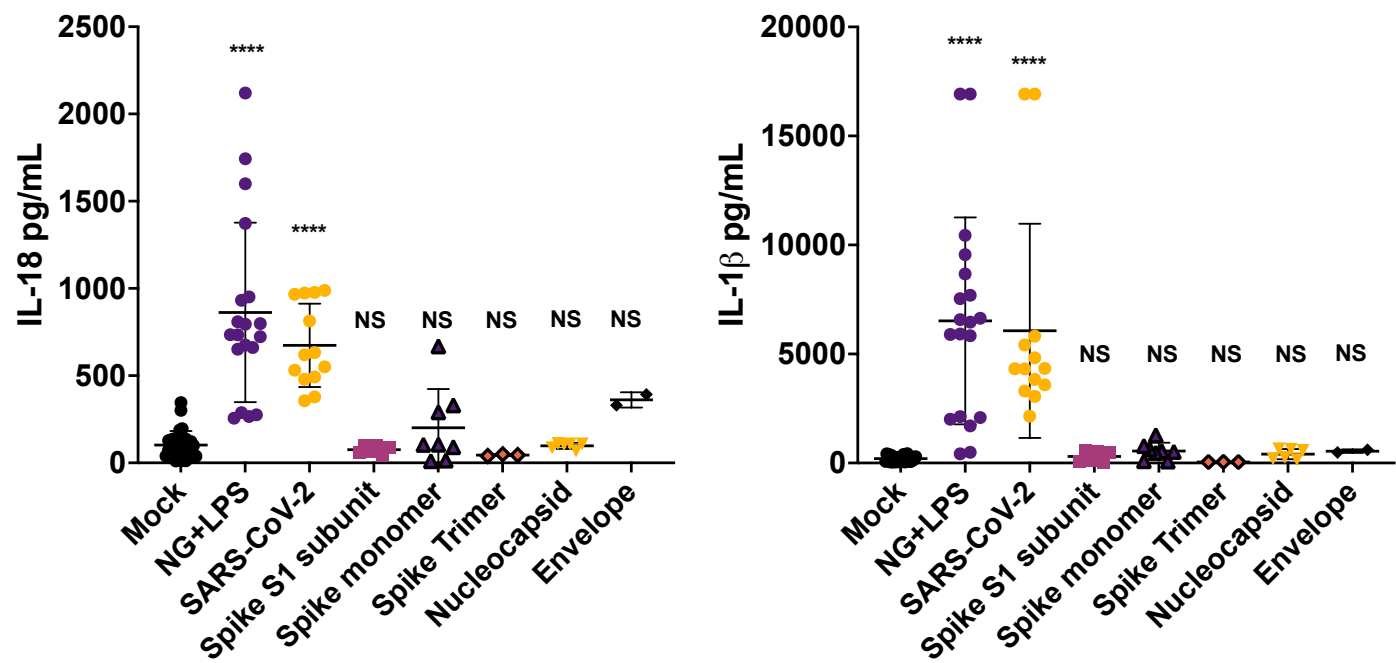

Supplement: Fig. S1 — LDH level, IL1B gene expression, and cytokine levels in THP-1 cells in response to SARS-COV-2 or recombinant structural proteins. [file mbio.00810-24-s0001.pdf]

A.

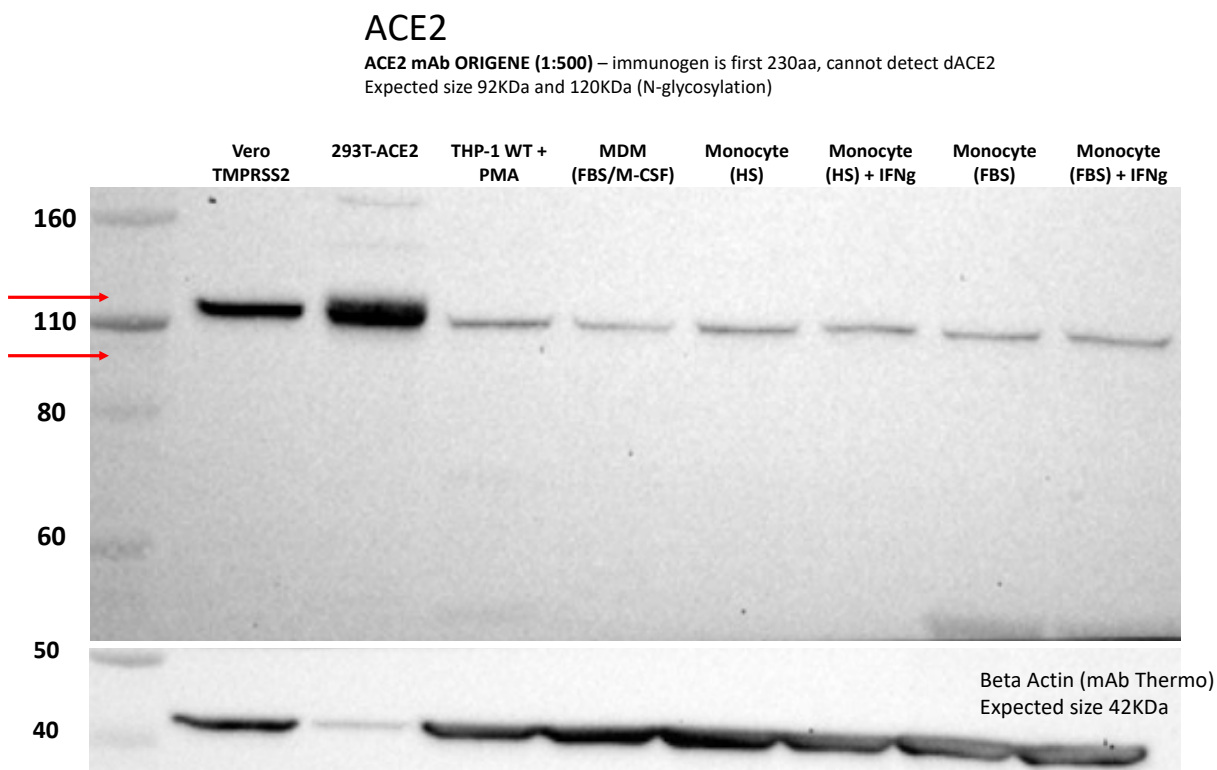

B.

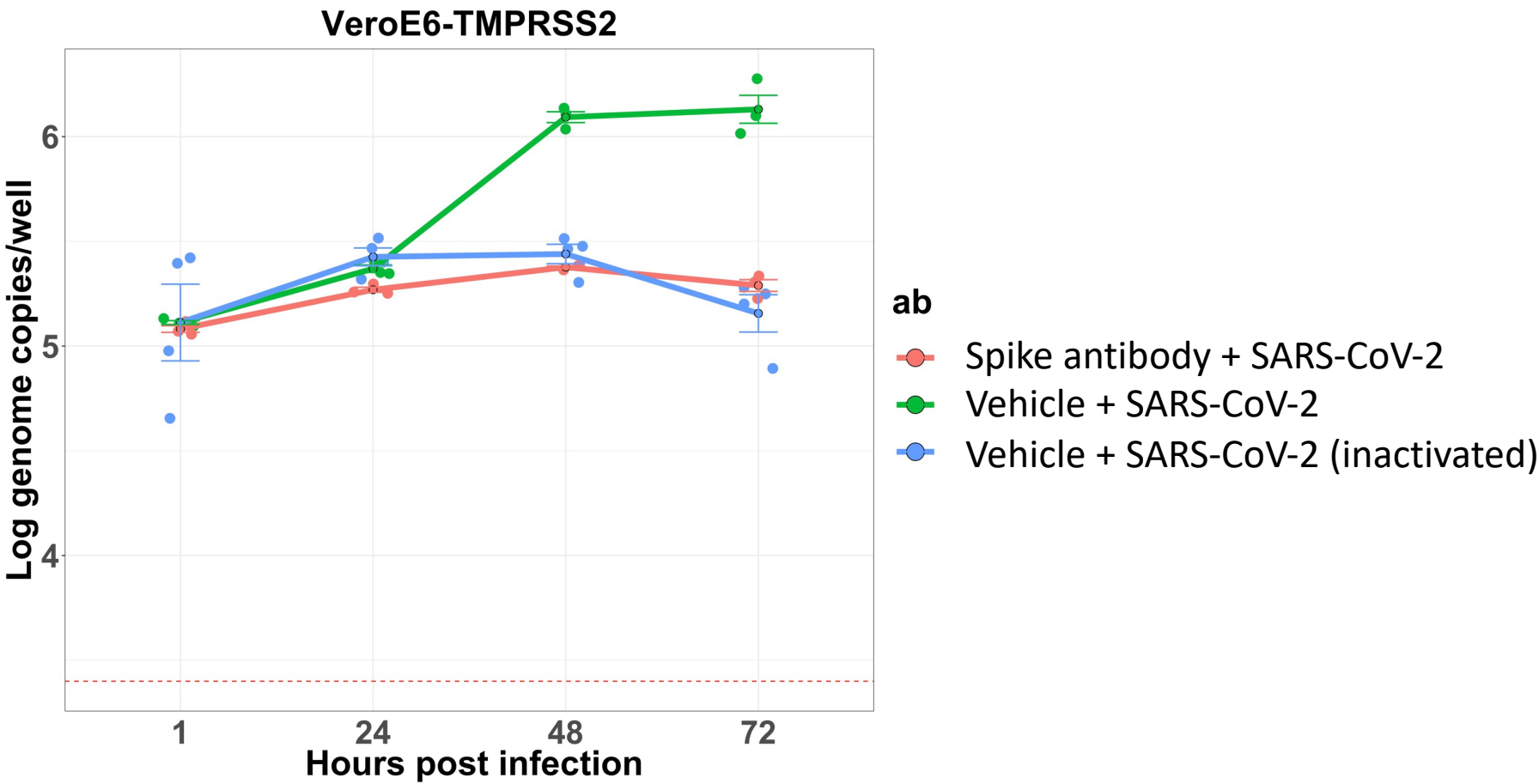

C.

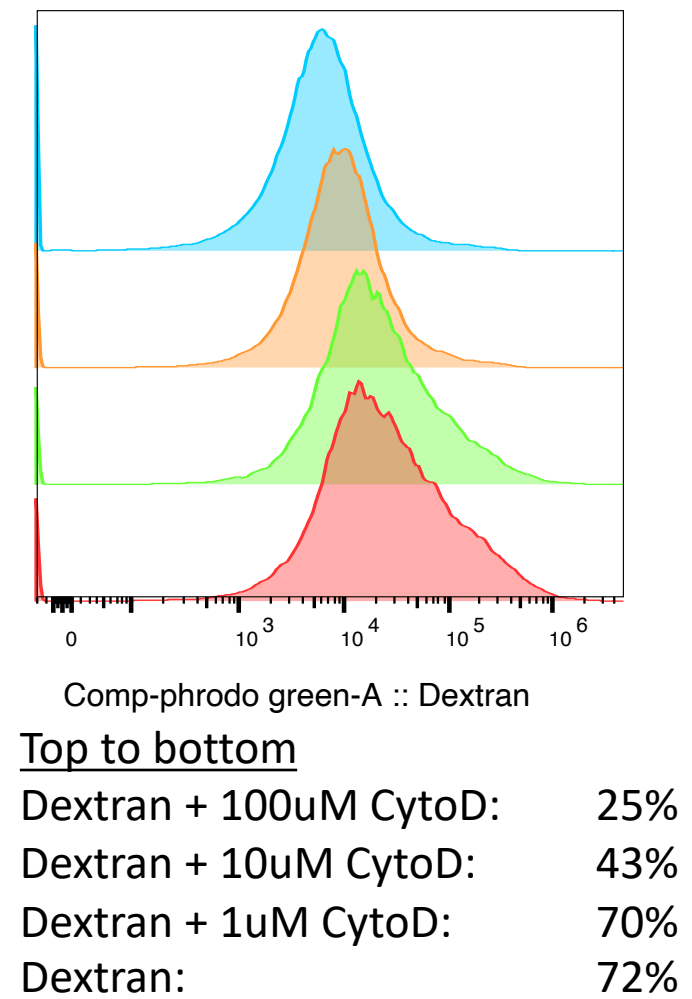

Supplement: Fig. S2 — Evaluation of cellular ACE-2 level, neutralizing spike antibody activity, and cytochalasin D activity in the indicated cell lines. [file mbio.00810-24-s0002.pdf]

Supplemental Fig 3

A.

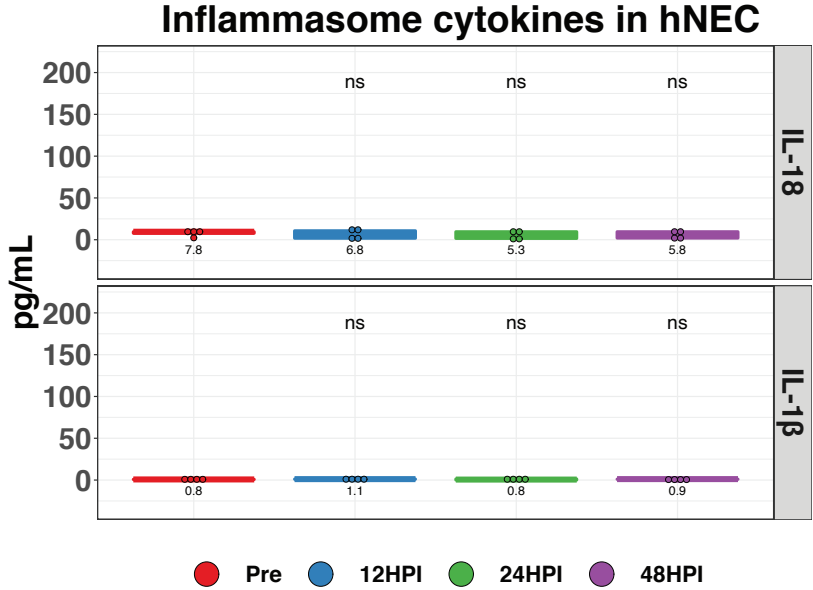

B.

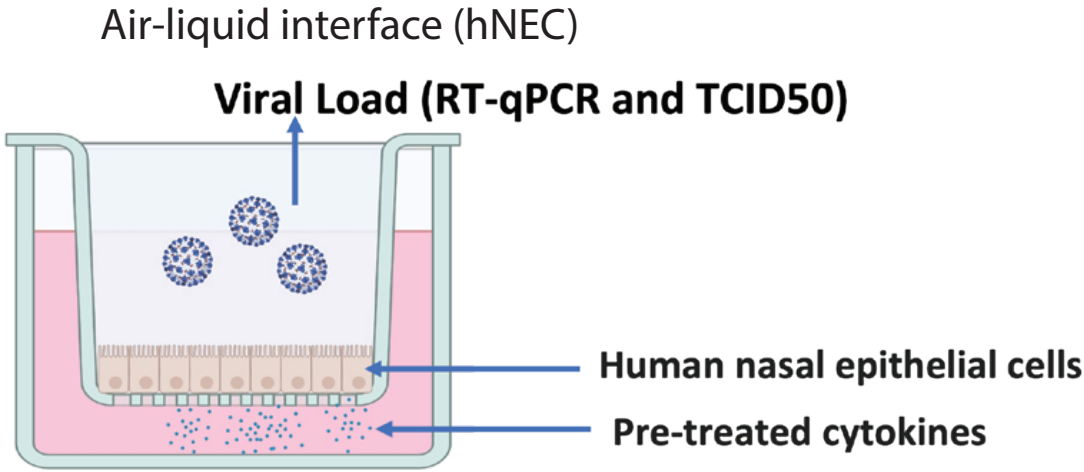

C.

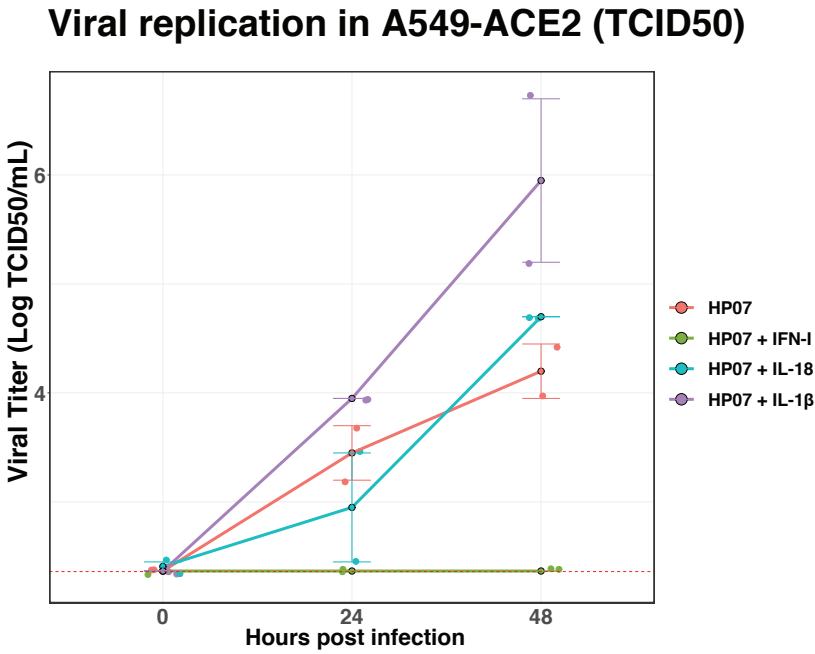

D.

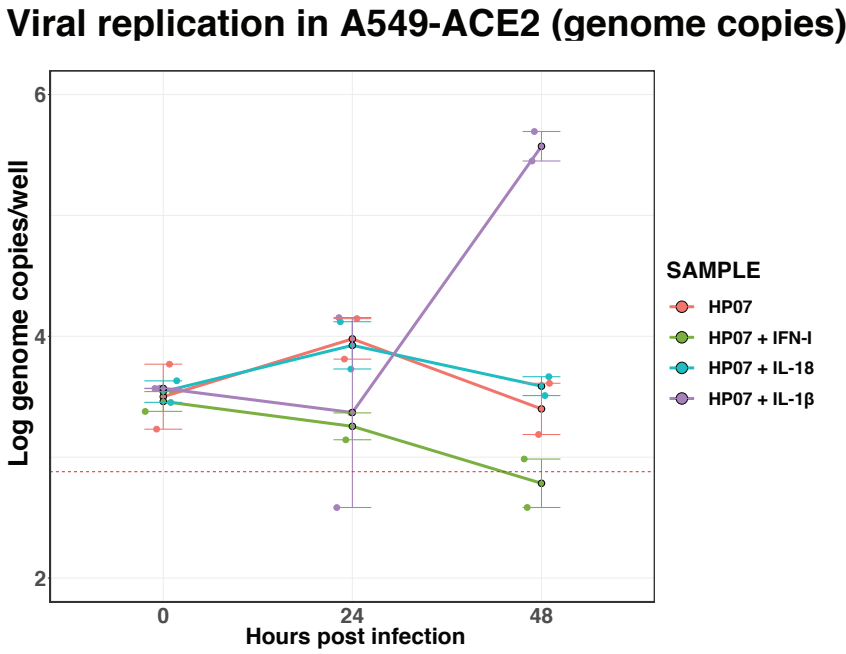

Supplement: Fig. S3 — SARS-CoV-2 viral load in A549-ACE2 cells with exogenous IL-18 and IL-1β treatment. [file mbio.00810-24-s0003.pdf]

Supplemental Fig 4

A.

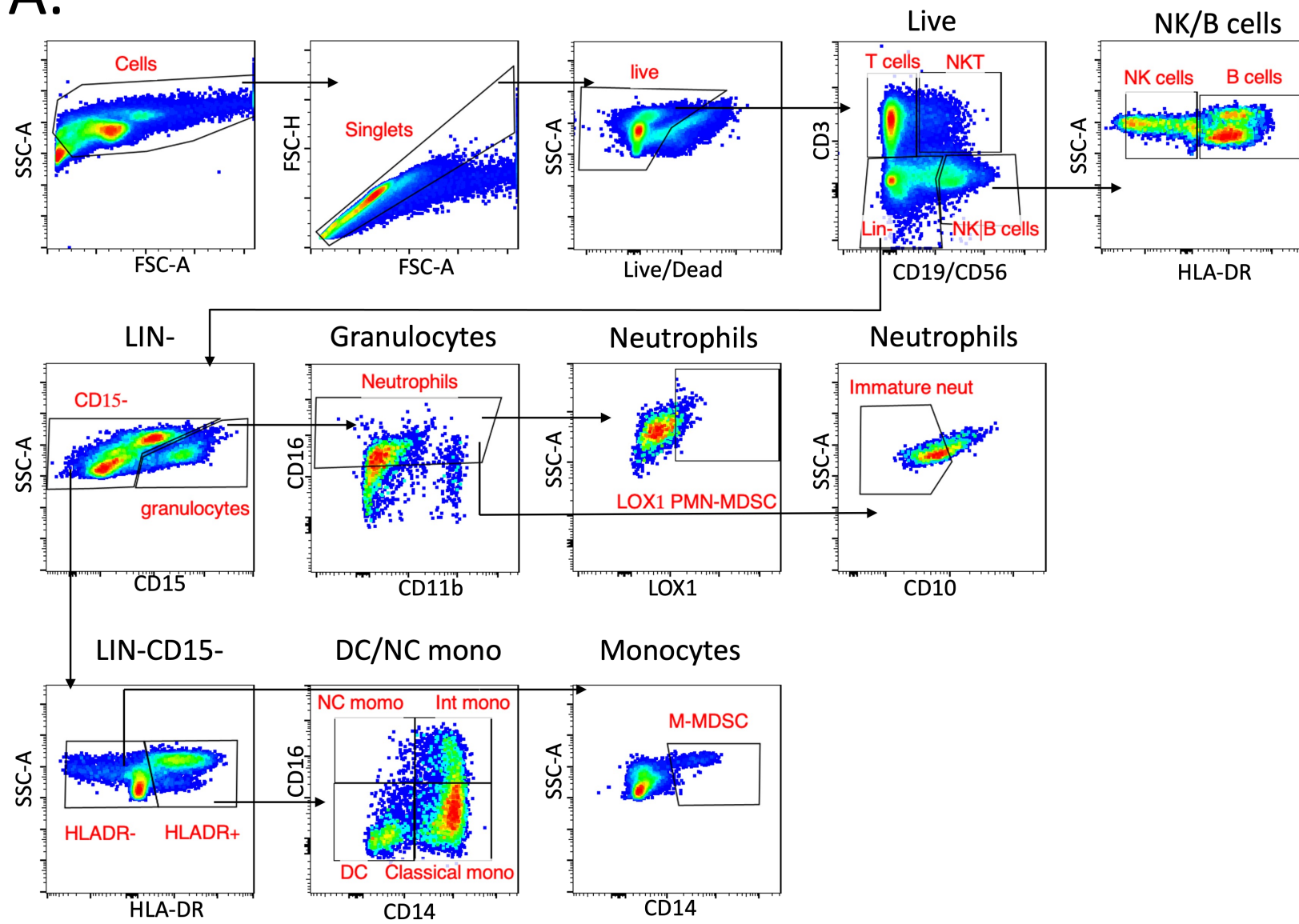

B.

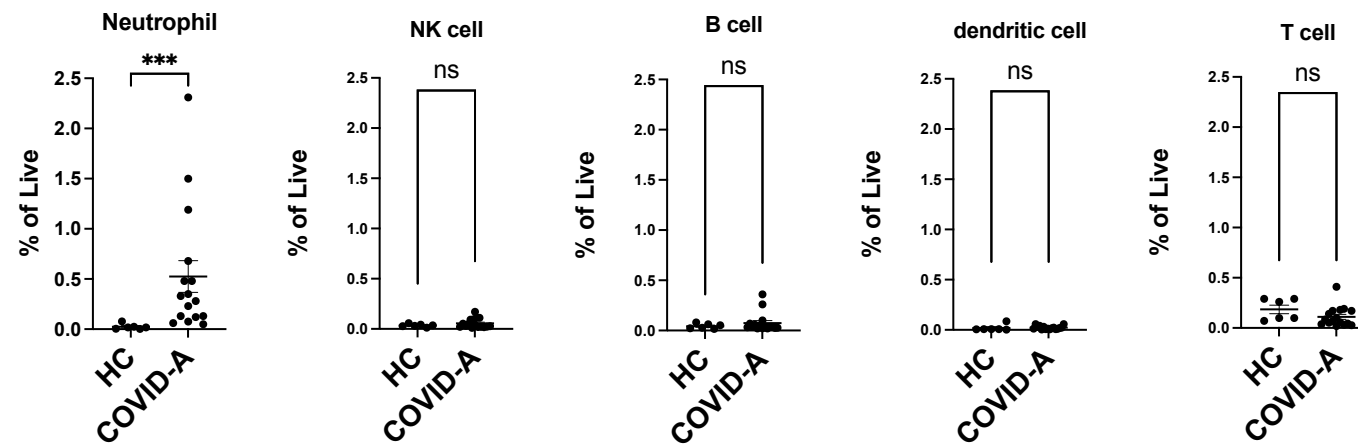

Supplement: Fig. S4 — PBMC gating strategy and PBMC population in healthy donors and COVID-19 patients. [file mbio.00810-24-s0004.pdf]
