# Supplementary material for: Bystander monocytic cells drive infection-independent NLRP3 inflammasome response to SARS-CoV-2
Source: mBio. 2024 Sep 6;15(10):e00810-24. doi: 10.1128/mbio.00810-24 (PMC11481483; doi:10.1128/mbio.00810-24)
Supplement: Fig. S5 — BAL sample gating strategy and monocyte population in COVID-19 patients. [file mbio.00810-24-s0005.pdf]

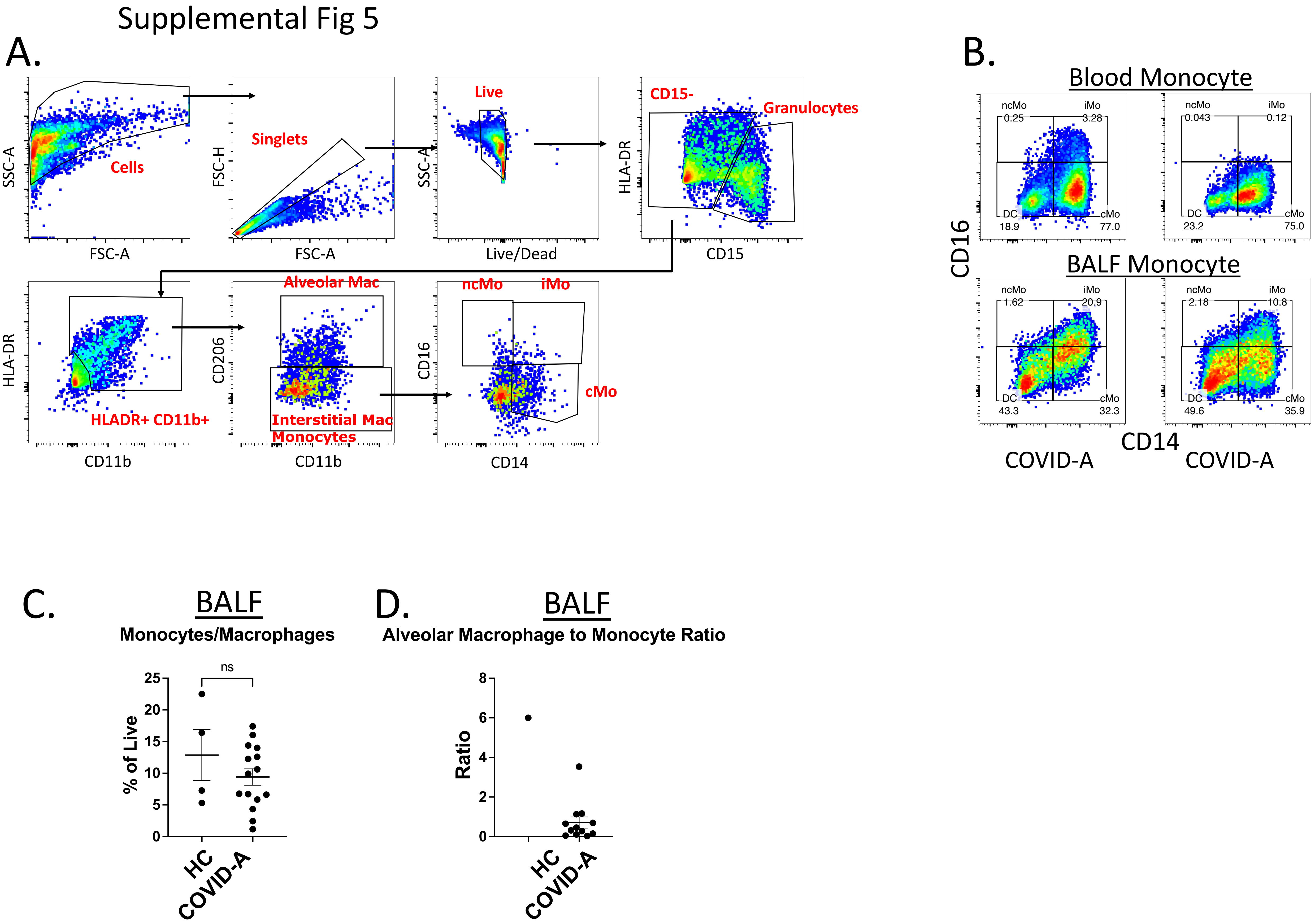
Supplemental Fig 5
A.
SSC-A
FSC-A
Cells
FSC-H
Singlets
Live
Live/Dead
HLA-DR
CD15-
Granulocytes
CD15
HLA-DR
CD11b
HLADR+ CD11b+
CD206
Alveolar Mac
Interstitial Mac
Monocytes
CD16
ncMo
iMo
cMo
CD14
B.
Blood Monocyte
BALF Monocyte
CD16
CD14
ncMo
0.25
iMo
3.28
DC
18.9
cMo
77.0
ncMo
0.043
iMo
0.12
DC
23.2
cMo
75.0
ncMo
1.62
iMo
20.9
DC
43.3
cMo
32.3
ncMo
2.18
iMo
10.8
DC
49.6
cMo
35.9
COVID-A
COVID-A
C.
BALF
Monocytes/Macrophages
% of Live
ns
HC
COVID-A
D.
BALF
Alveolar Macrophage to Monocyte Ratio
Ratio
HC
COVID-A
