# Supplementary material for: Bystander monocytic cells drive infection-independent NLRP3 inflammasome response to SARS-CoV-2
Source: mBio. 2024 Sep 6;15(10):e00810-24. doi: 10.1128/mbio.00810-24 (PMC11481483; doi:10.1128/mbio.00810-24)
Supplement: Supplemental legends — Legends for Fig. S1 to S5. [file mbio.00810-24-s0006.docx]

**Supplementary Figure 1.**

(A) LDH of THP-1 inoculated with mock, SARS-CoV-2, or NGLPS, measured 24-HPI. (B) LDH levels of WT, ΔAIM2, ΔASC, ΔCASP-1, and ΔNLRP3 THP-1 cells inoculated with mock, SARS-CoV-2, or NG+LPS, measured 24-HPI. Significance was tested using unpaired Mann-Whitney test compared with the indicated conditions. (C) IL1B gene expression fold change of indicated THP-1 cells inoculated with SARS-CoV-2 compared to mock. (D) THP-1 cells were inoculated with media alone (Mock), SARS-CoV-2 (MOI = 1), nigericin and lipopolysaccharide (NG+LPS), or the indicated recombinant SARS-CoV-2 structural proteins. Supernatants were collected 24-HPI and IL-18 (left panel), IL-1β (right panel) were measured by ELISA. Significance was tested using unpaired Kruskal-Wallis test with a Dunn's post hoc tests compared with mock condition.

**Supplementary Figure 2.**

(A) ACE-2 western blot of THP-1 cells and primary monocytes measured. Expected band for ACE2 is 120kDa. (B) SARS-CoV-2 genome copies in THP-1 cells inoculated with SARS-CoV-2 with or without spike specific neutralizing antibody or inoculated with inactivated SARS-CoV-2, measured at 1-, 24-, 48-, and 72-HPI. (C) Representative fluorescent intensity of Phrodo-green in THP-1s treated with Phrodo-green labled dextran and indicated concentration of Cytochalasin D (CytoD). Percentages indicate population with Phrodo-green signal.

**Supplementary Figure 3.**

(A) hNECs were inoculated with SARS-CoV-2 at MOI of 0.1. IL-18 (top) and IL-1β (bottom) levels of basolateral supernatant at pre-infection, 12-, 24-, and 48-HPI were measured by ELISA. (B) Graphical demonstration of the air-liquid interface hNEC model. Cytokines are added from the basolateral side, and cells are infected from the apical side. (C and D) A549 cell line overexpressing ACE2 were incubated with media (blue), type I Interferon (red), IL-1β (magenta), or IL-18 (green) in the basolateral media for 4 hours prior to infection with SARS-CoV-2. Inoculum were added for 1 hour. Supernatants were collected and viral load levels were measured by (C) RT-qPCR and (D) TCID50 at 24-, 48-HPI.

**Supplementary Figure 4.**

(A) Gating strategy of PBMC for study of monocytes. (C) Percentages of CASP1^+^ cells among neutrophils, NK cells, B cells, dendritic cells, and T cells. Significance was tested using unpaired Mann-Whitney test compared with the indicated conditions.

**Supplementary Figure 5.**

(A) Gating strategy of BAL fluid cells for study of monocytes. (B) Gating of CD14 CD16 comparing blood monocytes and BAL fluid monocytes. (C) Percentages of monocytic cells in HC and COVID-A. Significance was tested using unpaired Mann-Whitney test compared with the indicated conditions. (D) Ratio of alveolar macrophage to monocyte in HC and COVID-A.
