# Supplementary material for: Bystander monocytic cells drive infection-independent NLRP3 inflammasome response to SARS-CoV-2
Source: mBio. 2024 Sep 6;15(10):e00810-24. doi: 10.1128/mbio.00810-24 (PMC11481483; doi:10.1128/mbio.00810-24)
Supplement: Table S1 — Demographics of subjects studied. [file mbio.00810-24-s0007.docx]

**Supplementary Table 1.**

Demographics of subjects studied. Blood PBMC (top) and BAL fluid samples (Bottom).

*All subjects were not vaccinated or treated with dexamethasone

|  | COVID-19 | Healthy donor |
| --- | --- | --- |
| No. of subjects | 16 | 6 |
| Sex, male/female | 12/4 | 1/5 |
| Age, years (mean ± SD) | 57.25 ± 14.0 | 50.5 ± 11.84 |
| Severity range (WHO) | 1-7 | N/A |

|  | COVID-19 | Healthy donor |
| --- | --- | --- |
| No. of subjects | 9 (4 subjects with 2 collection dates during hospitalization) | 3 |
| Sex, male/female | 6/3 | 2/1 |
| Age, years (mean ± SD) | 62.55 ± 14.0 | 66.0 ± 9.0 |
| Severity range (WHO) | 7-8 | N/A |
